# The Gene Regulatory Cascade Linking Proneural Specification with Differentiation in *Drosophila* Sensory Neurons

**DOI:** 10.1371/journal.pbio.1000568

**Published:** 2011-01-04

**Authors:** Sebastián Cachero, T. Ian Simpson, Petra I. zur Lage, Lina Ma, Fay G. Newton, Eimear E. Holohan, J. Douglas Armstrong, Andrew P. Jarman

**Affiliations:** Centre for Integrative Physiology, School of Biomedical Sciences, University of Edinburgh, Edinburgh, United Kingdom; Stanford University, United States of America

## Abstract

In neurogenesis, neural cell fate specification is generally triggered by proneural transcription factors. Whilst the role of proneural factors in fate specification is well studied, the link between neural specification and the cellular pathways that ultimately must be activated to construct specialised neurons is usually obscure. High-resolution temporal profiling of gene expression reveals the events downstream of *atonal* proneural gene function during the development of *Drosophila* chordotonal (mechanosensory) neurons. Among other findings, this reveals the onset of expression of genes required for construction of the ciliary dendrite, a key specialisation of mechanosensory neurons. We determine that *atonal* activates this cellular differentiation pathway in several ways. Firstly, *atonal* directly regulates *Rfx*, a well-known highly conserved ciliogenesis transcriptional regulator. Unexpectedly, differences in *Rfx* regulation by proneural factors may underlie variations in ciliary dendrite specialisation in different sensory neuronal lineages. In contrast, *fd3F* encodes a novel forkhead family transcription factor that is exclusively expressed in differentiating chordotonal neurons. *fd3F* regulates genes required for specialized aspects of chordotonal dendrite physiology. In addition to these intermediate transcriptional regulators, we show that *atonal* directly regulates a novel gene, *dilatory*, that is directly associated with ciliogenesis during neuronal differentiation. Our analysis demonstrates how early cell fate specification factors can regulate structural and physiological differentiation of neuronal cell types. It also suggests a model for how subtype differentiation in different neuronal lineages may be regulated by different proneural factors. In addition, it provides a paradigm for how transcriptional regulation may modulate the ciliogenesis pathway to give rise to structurally and functionally specialised ciliary dendrites.

## Abstract 2

Temporal expression profiling of sensory precursor cells reveals how the *atonal* proneural transcription factor regulates a specialized neuronal differentiation pathway.

## Author Summary

Early during development, cells differentiate and take on specialized forms and functions. This requires the activation of specific genes for different cellular pathways. Our study addresses how this activation is regulated in the developing *Drosophila* nervous system. In this model, it is well known that proneural transcription factors are involved in directing cells to differentiate into various types of neurons. However, the mechanism by which they choreograph the activation of genes for neuronal differentiation is not clear. In this study, we focused on events leading to differentiation of mechanosensory neurons, which have specialized dendritic processes that mediate sensory perception. In these developing neurons we profiled the time course of gene expression that is triggered by the proneural factor *atonal*. Our analysis revealed the activation of genes required for the formation of these specialized dendrites, called cilia. We then identified several ways in which *atonal* regulated these genes. First, it activates intermediate transcription factors that regulate different subsets of differentiation genes. Second, in at least one case, *atonal* activates a differentiation gene directly, one that is involved in the formation of cilia (ciliogenesis). These findings offer new insight into how proneural factors regulate specialized neuronal differentiation pathways.

## Introduction

Once an embryonic cell is committed to a particular fate, it is likely that a precisely ordered progression of gene expression is required to coordinate the complex cell biological events that eventually lead to its terminal differentiation. Determining how this progression is regulated is an important step towards understanding how cells acquire specialised morphologies and functions. In the developing nervous system, cell fate commitment is initiated by the activity of proneural basic-helix-loop-helix (bHLH) transcription factors [1]. In vertebrates, *atonal* (*ato*)-related proneural genes are required for neurogenesis in the spinal cord and cortex (*neurogenin*), cerebellum (*atoh1*), and retina (*atoh7*) [1]. *atoh1* is also required for the formation of mechanosensory cells in the inner ear and in skin [2],[3]. In *Drosophila*, *ato* itself specifies the precursors of several specialised sensory neuron types, including photoreceptors and mechanosensory chordotonal (Ch) neurons, which mediate hearing and proprioceptive feedback during locomotion [4]. Whilst proneural genes are intensively studied, little is known of how their function leads to specific programs of neuronal differentiation.

**Figure 1:**
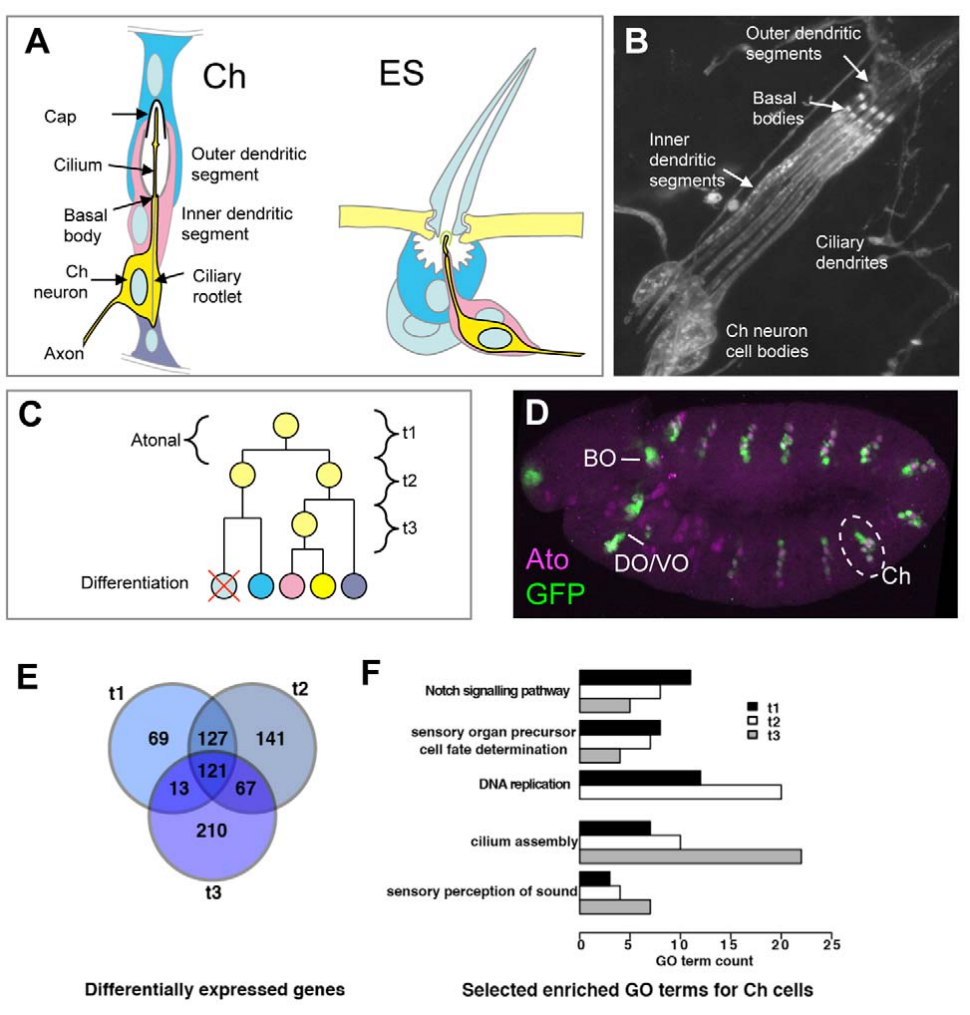
Gene expression profiling of Ch cells. (A) Schematic of structural features of Ch and ES organs. (B) Group of five Ch neurons in the larval lateral body wall, labelled with anti-HRP, which detects the cell body and inner dendritic segment. The approximate location of the basal body is indicated. (C) Schematic of cell lineage leading from an SOP to a Ch organ (same colour scheme as in A). Ato is expressed at the SOP stage. The time points sampled for analysis are indicated approximately (t1, t2, t3). (D) Stage 11 embryo expressing *ato*GFP. GFP (green) and *ato* protein (magenta) are co-expressed in Ch precursors in the trunk. GFP fluorescence is also detected in several *ato*-dependent head sense organs, including Bolwig's Organ (BO), Dorsal Organ (DO), and Ventral Organ (VO). (E) Venn diagram of genes enriched in *ato*GFP cells at three developmental time points, representing the first 3 h of Ch cell development. Genes shown are enriched in *ato*GFP+ versus *ato*GFP− cells (≥1.5-fold, 1% FDR). (F) Developmental profiling of gene expression in *ato*GFP. Bars represent the number of genes associated with selected GO terms. GO terms associated with early development (‘Notch signalling pathway’, ‘sensory organ precursor cell fate determination’) decrease from t1 to t3. Conversely, the differentiation terms ‘cilium assembly’ and ‘sensory perception of sound’ increase progressively. Terms shown are all significantly enriched (Tables S4, S5, S6, S8).

*ato* expression in the ectoderm leads to sense organ precursor (SOP) specification in a process that is refined by Notch signalling. After commitment, SOPs divide several times asymmetrically before the 4–5 progeny cells interact and terminally differentiate to form the neuron and support cells of the mature Ch sense organ (Figure 1A–C). The function of *ato* and other proneural factors in SOP fate determination is relatively well studied. Indeed, known proneural target genes are almost all concerned with SOP specification or fate maintenance [5]–[9]. It is not clear, however, how its function as ‘master regulator’ leads to subsequent neural development. Since *ato* is expressed only transiently during SOP formation, a likely hypothesis is that it initiates a gene regulatory cascade, which eventually regulates differentiation genes. The nature of this cascade and its regulation have not been elucidated.

In contrast to the dearth of knowledge of the regulatory cascade, more is known of Ch neuron terminal differentiation itself. Notably, Ch neurons develop a highly structured dendrite based on a modified cilium [10]–[12]. Ciliogenesis is a conserved, highly ordered process involving the coordinated action of hundreds of proteins [13]. In vertebrates, ciliated cells are widespread, both in the PNS (e.g. photoreceptors, olfactory neurons) and other adult tissues (e.g. kidney, lung), and developing cells have a primary cilium that is required for signal transduction for a number of paracrine pathways [13]. In contrast, the only ciliated cells in *Drosophila* are sensory neurons and sperm. As a consequence, genetic analysis of defective sensory neuron differentiation in *Drosophila* has enabled the discovery and characterisation of a number of ciliogenesis genes [14]–[16]. These include genes required for the specialised transport process known as Intraflagellar Transport (IFT) [16] and homologues of genes disrupted in the human ciliopathy, Bardet-Biedl syndrome (BBS). Ciliogenesis is one of the key differentiation events that must be initiated by *Drosophila* proneural factors.

An important question in the regulation of cellular diversity is how core cell biological pathways are modified to give distinct cell types. Cilia perform a wide variety of specialised functions, but it is poorly known how the core ciliogenesis program is modulated in different cell types. The ciliary dendrite of Ch neurons is anatomically and physiologically distinct from those of other *Drosophila* sensory neurons (notably the External Sensory (ES) neurons) (Figure 1A) [17],[18]. Ultimately, these subtype-specific differences in the ciliary dendrite must be regulated by the proneural factors, which have well-known neuronal subtype determining properties in both invertebrates and vertebrates [1]. Whilst *ato* directs the formation of Ch precursors, another proneural gene, *scute* (*sc*), performs this function for ES precursors. *sc*'s function is likely to be mediated partly by the homeodomain factor, Cut, [19] but little is known of Cut's molecular function. Apart from the involvement of *cut*, it is at present entirely unknown how subtype specification by transiently expressed proneural factors is translated into differences in neuronal phenotype, including the modulation of ciliogenesis.

In order to bridge the gap between proneural factor function and the activation of genes required for neural terminal differentiation, we used expression profiling to characterise the progression of gene expression during Ch neuron development. A time course in the onset of differentiation gene expression can be discerned. We then show that *ato* regulates some of these events through a number of intermediate transcriptional regulators. The gene for Regulatory factor X (*Rfx*), a well-known and highly conserved regulator of aspects of ciliogenesis, is regulated differently by proneural genes in Ch and ES lineages. We propose that this links proneural subtype specification to differences in ciliogenesis. We also identify a novel forkhead-related factor that is required to regulate genes for specialised aspects of Ch neuron function. In addition, we find that some putative differentiation genes are expressed surprisingly early in neural development and that *ato* may directly regulate at least one such gene.

## Results

### High-Resolution Expression Profiling of Embryonic Ch Cells

For expression profiling during Ch development, *ato*-expressing cells were isolated from timed collections of embryos. *ato*-expressing cells were marked by GFP expression from an *ato*GFP reporter gene construct (*ato*GFP cells). This reporter gene is expressed predominantly in Ch precursors and their progeny but also in other *ato*-expressing cells including the developing larval eye (Figure 1D). Embryos from timed collections were dissociated and *ato*GFP cells isolated by FACS (Figure S1). Such cells were isolated from embryos at three time points corresponding to the first 3 h of neural development (t1–t3) (Text S1). t1 coincides maximally with *ato* expression (and therefore should include direct target genes), whereas later time points reflect subsequent post-*ato* development as the precursors divide leading up to differentiation (Figure 1C).

**Figure 2:**
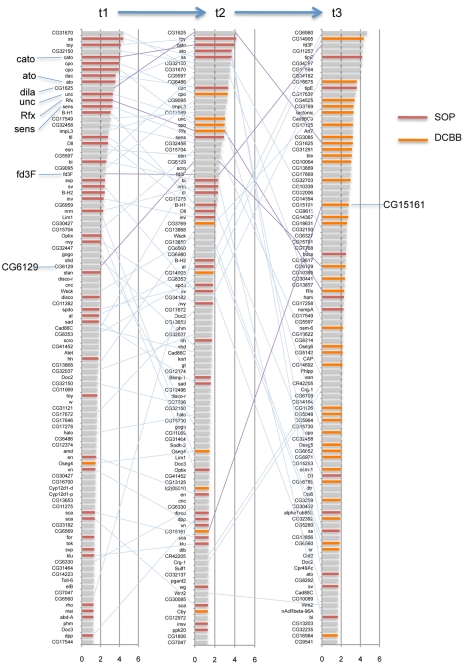
The progression of gene expression in Ch neurons. The top-ranked 100 genes are shown for each time point with bars representing log_2_(fold change). Some of the genes mentioned in this study are highlighted. Genes with previous evidence of function or expression in PNS development are indicated with red bars; those present in the *Drosophila* Cilia and Basal Body database (and therefore linked to cilium development or function) are in orange.

Expression profiling revealed the number of differentially expressed genes in *ato*GFP+ versus *ato*GFP− cells (referred to as ‘*ato*-correlated genes’) to be 330, 456, and 411 at t1, t2, and t3, respectively (≥1.5-fold enriched, ≤1% false discovery rate (FDR)). Set analysis of genes enriched in *ato*GFP cells (*ato*-correlated genes) shows a clear time course of expression changes, with 69, 141, and 210 genes unique to t1, t2, and t3, respectively (Figure 1E; Tables S1, S2, S3). This suggests an increase in the complexity of gene expression as development proceeds to differentiation. Manual inspection of *ato*-correlated genes ranked by fold change shows a high representation of known neurogenesis genes (Figure 2; Tables S1, S2, S3). For instance, among the top ranked genes at t1 are *spineless*, *twin of eyeless*, *cato*, *couch potato*, *dachshund*, *ato*, *Rfx*, *senseless*, and *BarH1*, all of which are associated with aspects of neural development. Gene ontology (GO) analysis shows strong enrichment of GO annotation terms related to PNS development across all three time points (Tables S4, S5, S6, S7; Text S2). There is a clear progression over time in the representation of genes (Figure 2) and relevant GO terms (Figure 1F; Text S2).

### Genes Implicated in Ciliogenesis Are Strongly Represented in Developing *ato*GFP Cells

Over-representation of GO terms identified the enrichment in developing Ch cells of genes associated with ciliogenesis (Text S3; Figure 1F). Analysis of over-represented protein domains also highlights domains associated with some classes of ciliogenesis gene (Text S4; Figure S2; Table S8). To characterise the Ch expression of ciliogenesis genes further, we compared the t3 expression data with collections of genes previously linked to ciliogenesis (Tables 1, S9). The *Drosophila* Cilia and Basal Body database (DCBB) has been compiled from a number of genetic and proteomic sources to contain genes, or orthologues of genes, implicated in cilia or basal body structure or function [20]. *ato*-correlated genes at t3 represent 3.0% of the genome but include 10.1% of DCBB genes—a highly significant over-representation (*p* = 3.3×10^−19^) (Table 1). Another study identified potential ciliogenesis genes from comparative genomic analysis of ciliated and non-ciliated organisms [16]. Strikingly, Ch cells at t3 are 8-fold more enriched than expected by chance for genes implicated in this study (*p* = 8.7×10^−23^) (Table 1). Moreover, the subgroup of these genes most associated with compartmentalised ciliogenesis are 27-fold enriched at t3 compared with expected (*p* = 3.5×10^−22^) (23/28 genes) (Table 1). For many of these genes, our expression data provide the first confirmatory evidence of a potential role in ciliogenesis. Our data also provide new candidate ciliogenesis genes.

**Table 1:** Summary of differentially expressed genes in *ato*GFP cells particularly in relation to ciliogenesis^1^ (see also Table S9).

Gene Group	Total	t1	t2	t3
All genes (on microarray)	14,075	341 (2.4%)	487 (3.5%)	429 (3.0%)
Genes associated with ciliogenesis^2^	174	15* (8.6%)	18* (10.3%)	42* (24.1%)
Compartmental subset	28	6* (21.4%)	8* (28.6%)	23* (82.1%)
*Drosophila* cilium and basal body database (DCBB) [20]	750	32* (4.3%)	44* (5.9%)	76* (10.1%)
Genes with conserved X box motif^3^				
Stringent match	83	7* (8.4%)	12* (14.5%)	18* (21.7%)
Looser match	384	21* (5.5%)	26* (6.8%)	40* (10.4%)
Proneural cluster genes^4^	197	25* (12.7%)	33* (16.8%)	21* (10.7%)

1 This analysis uses 1.5-fold enriched, 1% FDR, trusted genes only. Percentages refer to the proportion of genes in that group that are differentially expressed at each time point. Figures with asterisk are significantly over-represented as determined by Fisher exact test (*p*<0.05).

2 Genes associated with ciliogenesis are derived from a comparative genomic analysis of ciliated and non-ciliated organisms [16]. The compartmental subset contains those associated with compartmentalised ciliogenesis that have few ESTs (i.e., are rare transcripts) and have a nearby X box motif (see also Table S9).

3 Data for genes with conserved X boxes were taken from [20].

4 Proneural cluster genes: previous expression profiling of genes expressed in ES proneural cluster cells in wing imaginal discs (see Text S5 for details) [55].

Since the *ato*GFP cells will divide to produce both the Ch neurons and their support cells, *ato*-correlated genes may include support cell genes in addition to neuronal genes. Few such genes are currently known, but several of these are enriched at t3 (but not earlier), including *nompA* (scolopale cell) [21], *α-tubulin 85E* (ligament and cap cells) [22], and *Sox15* (cap cell) [23].

### Temporal Sequence of Differentiation Gene Expression

**Figure 3:**
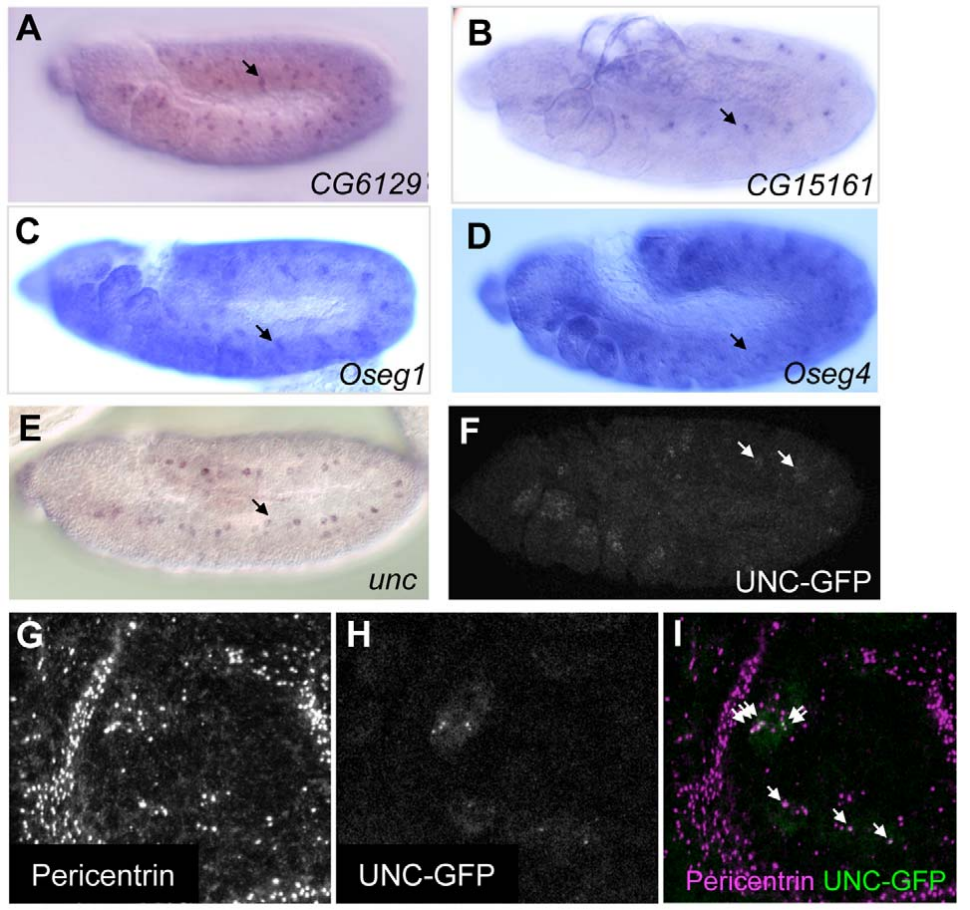
Many differentiation genes are expressed at the neural precursor stage. (A–E) Stage 11 embryos showing early mRNA expression of several ciliogenesis genes. Arrows point to sensory precursor cells or their direct progeny. (A) *CG6129* (rootletin homologue). (B) *CG15161* (IFT46 homologue). (C) *Oseg1* (IFT122 homologue). (D) *Oseg4* (WDR35 homologue). (E) *unc* (basal body protein). (F–I) Expression and localisation of an UNC-GFP fusion protein from a construct in which the *unc* promotor and ORF are fused to GFP [14]. (F) Stage 11 embryo. UNC-GFP is expressed in sensory precursor cells (arrows). (G–I) Magnification of one segment from (F). UNC-GFP colocalises with the centrosome marker, Pericentrin, in a subset of cells—the Ch precursors. At later stages UNC-GFP localises to the basal body of the ciliary dendrite (unpublished data).

It is striking that our analyses indicate enrichment for genes required for ciliary differentiation, because terminal Ch differentiation has not yet occurred by the embryonic stage represented at t3 (approximately Stage 12). This suggests that some aspects of differentiation require the activation of specific differentiation genes prior to overt differentiation. Unexpectedly, a proportion of ciliary genes are already expressed even at t1 (8.6% of all ciliogenesis genes (14/175), 21.4% of compartmentalised ciliogenesis genes (6/28); Table 1). At t1 the Ch precursor cells have just been specified by *ato* and have still to undergo two rounds of division before neuronal differentiation occurs. In situ hybridisation confirmed that mRNAs for several ciliogenesis genes are expressed in Ch precursors or in their first division products. This includes genes required for a wide range of cilia components, such as the ciliary rootlet (*CG6129* – homologue of Rootletin), the IFT-B complex (*CG15161* – homologue of IFT46), and the IFT-A complex (*Oseg1* – homologue of IFT122; *Oseg4* – homologue of WDR35) (Figure 3A–D). Most striking, for instance, is *unc*, which is thought to be involved in basal body maturation [14]. Although reported to be expressed only upon differentiation [14], we find that *unc* RNA is already 9.9-fold enriched at t1 (ranked 11^th^), and early expression is confirmed by in situ hybridisation (Figure 3E). Furthermore, UNC protein is also expressed early and is already localised to the centrosomes in Ch precursor cells (Figure 3F–I). Conversely, many known differentiation genes are not differentially expressed even at t3, supporting the conclusion that general differentiation has not yet occurred. This includes the Ch-specific TRPV-encoding genes, *nanchung* (*nan*) and *inactive* (*iav*), sensory neuron genes like *futsch* (MAP1B), and several groups of ciliogenesis gene. Therefore, a specific progression of gene expression can be discerned that defines a temporal program for organised ciliogenesis and neuronal differentiation.

### Expression Analysis of Enriched Genes Identifies a Characteristic Ch-Enriched Pattern

A precise program of gene activation implies that transcriptional regulation is important for coordinating the cell biological events underlying ciliogenesis, yet little is known of the gene network underlying this. As a first step in exploring the transcriptional regulation of Ch genes, we characterised expression patterns of a sample of *ato*-correlated genes by in situ hybridisation (Table S10; sample chosen based on fold change and lack of previous detailed annotation relating to PNS expression pattern). At least 90% of genes tested (*n* = 43) showed expression patterns that overlap *ato*-expressing cells, and the vast majority of these showed expression in Ch cells (Figure S3). Moreover, most of these genes showed expression in the neuronal branch of the sensory lineage, rather than in support cells. Given the nature of the profiling (Ch cells compared with the rest of the embryo), we expected expression in *ato*-dependent cells, but not necessarily restricted to such cells within the nervous system. Indeed, various types of pattern were observed, including those we categorise as pan-neural (CNS and PNS), pan-sensory (PNS only), or Ch-specific. This distribution of patterns is broadly consistent with the view that the related Ch and ES lineages have both shared and unique properties. Unexpectedly, however, a significant proportion of genes show an intermediate ‘Ch-enriched’ pattern, characterised by strong and early onset expression in the Ch lineage but weak and later onset in the ES lineage (Figure S3). This includes many differentiation and ciliogenesis genes (including those mentioned above) that might otherwise have been expected to be required equally in all ciliated sensory lineages (pan-sensory). We suggest therefore that the subtype differences between the two main neuronal lineages with ciliary dendrites (Ch and ES) may partly arise from modulation in timing and level of expression of genes required for a common cellular differentiation program.

Since *ato/sc* proneural genes control the acquisition of Ch/ES subtype identity [24], the modulation of differentiation suggested above must ultimately result from differences in proneural gene function. In order to link the regulation of differentiation to *ato* function, we carried out profiling of *ato*-expressing cells from *ato* mutant embryos at t1. In such embryos, *ato*GFP-expressing cells largely fail to become specified as Ch precursors and remain as ectodermal cells. Comparison with the wildtype expression profile yields 50 genes that are ≥2-fold differentially expressed in wild-type *ato*GFP+ cells at t1 (compared with the GFP– cells) but not in mutant *ato*GFP+ cells (compared with the GFP− cells from the same embryos) (Table S11). Of these, 11 genes also show a ≥2-fold difference between the fold changes observed in wildtype and mutant embryos, which represent good candidates for downstream targets (Table S12). Three of these encode transcription factors (*Rfx*, *cato*, and *fd3F*). These genes were investigated as candidate intermediate regulatory factors that link proneural function to differentiation.

### A Regulator of Ciliogenesis, RFX, Is Regulated by *ato* and *sc* in Different Ways

RFX is a well-known, highly conserved regulator of ciliogenesis and is best known as a proven or predicted regulator of many ciliogenesis genes through binding to an X-box motif (notably those genes associated with IFT-B) [20],[25]. Although required for neuronal differentiation, the *Rfx* gene is already highly expressed in the earliest *ato*GFP cells (9.76-fold enriched at t1, ranked 12^th^), indicating that it may be responsible for early expression onset of a subset of differentiation genes. Consistent with this, a resampling analysis demonstrates that gene lists for all three time-points are highly significantly enriched for the presence of nearby X box motifs (Figure S4), indicating the likely presence of *Rfx* target genes. In addition, of the set of 83 genes in the genome that have a conserved perfect X box motif nearby [20], 21.7% are expressed at t3—a 7.1-fold greater frequency than expected by chance (*p* = 8.23×10^−10^) (Tables 1, S9). These include ciliogenesis genes for which experimental evidence has been obtained that they are direct *Rfx* targets (such as *CG15161*, *btv*, *tectonic*, *CG6129*, *CG4525*) [20].

**Figure 4:**
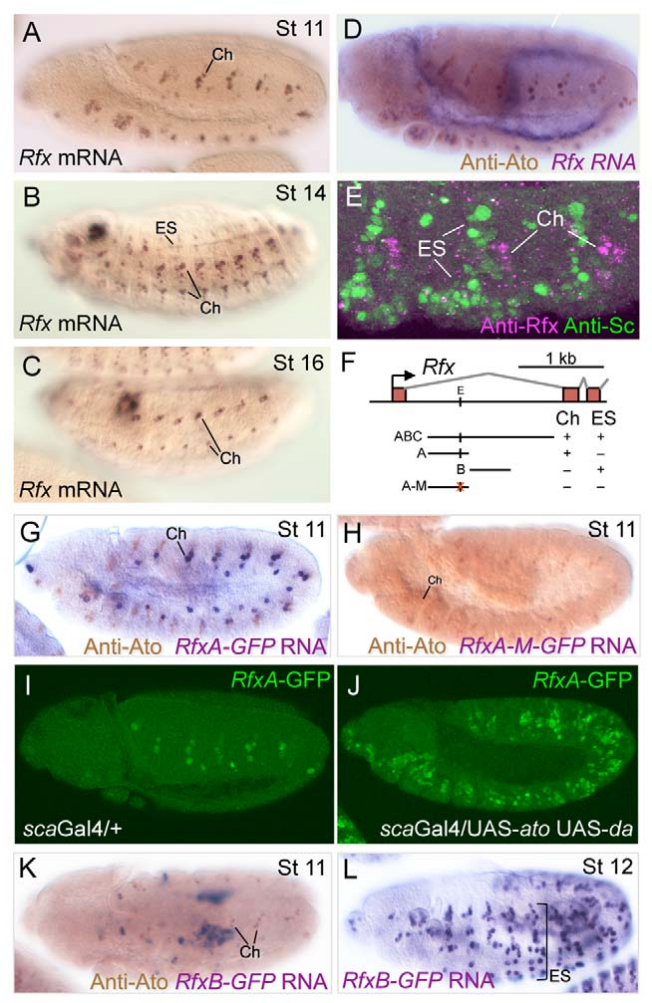
*Rfx* is a Ch-enriched gene that is directly regulated by *ato*. (A–C) *Rfx* is expressed in a ‘Ch-enriched’ pattern despite being required for both Ch and ES ciliary differentiation. (A) Early in neurogenesis, *Rfx* protein is present in Ch precursors but not ES precursors. (B) Later expression is strong in Ch lineages and weak in ES lineages. (C) During differentiation, Rfx protein is largely confined to Ch neurons. (D) Co-expression of *Rfx* mRNA and *ato* protein in Ch precursor cells. (E) Three segments from embryo stained to detect *Rfx* (magenta) and *sc* (green) proteins. There is no expression of *Rfx* in *sc*-expressing ES precursor cells. (F) Schematic of first three exons of *Rfx* gene, showing the location of separate Ch and ES enhancers; the tested E box is indicated (‘E’); lines indicate fragments tested in GFP reporter assay, with a summary of their expression. (G) GFP driven by *RfxA* enhancer is expressed early in Ch lineages. GFP mRNA is coexpressed with *ato* protein. (H) Mutation of an E_ATO_ box in *RfxA* abolishes the early Ch expression of GFP; Ch cells are marked by *ato* expression. (I,J) *RfxA*-GFP is ectopically expressed in response to *ato* misexpression. (I) Expression of *RfxA*-GFP in *sca*Gal4 driver background (wild type). (J) Ectopic expression of *RfxA*-GFP in embryo in which *ato* protein and its dimerisation partner, *daughterless* (*da*), are jointly misexpressed in the ectoderm. (It has been shown that proneural factor activity in embryos is limited by *da* levels such that misexpression of a proneural factor alone has little effect [53]). (K,L) GFP driven by *RfxB* is not expressed at stage 11 (when ES and Ch precursors are present) (K) but is expressed later in ES lineages (L). We note that the *RfxB* enhancer also contains an E_ATO_ motif even though the enhancer is not active in Ch lineages; however, we cannot rule out the possibility that this motif is a functional ATO binding site in the context of the intact *Rfx* locus.

Although *Rfx* is required for both Ch and ES neurons, examination of its expression pattern revealed that, like many of its target genes, it shows a Ch-enriched pattern of expression (Figure 4A–C). It is possible, therefore, that variations in *Rfx* expression may underlie different subtype-specific programs in Ch and ES cells. In turn, this suggests that *Rfx* may be regulated differently by ATO and SC proteins in these lineages as part of their neuronal subtype-determining function. Therefore, we examined the regulation of *Rfx* by proneural factors. Embryonic expression analysis confirmed that Ch expression of *Rfx* overlaps with that of *ato* (Figure 4D). In contrast, *Rfx* expression in ES lineages begins later, only after the termination of *sc* expression (Figure 4E). By reporter gene analysis, we found that *Rfx* is regulated through separable Ch and ES enhancers (Figure 4F). The Ch enhancer is activated early in Ch development (*RfxA*: Figure 4G). This enhancer contains an E box motif whose sequence conforms to that previously shown to respond specifically to ATO activation (E_ATO_) [7]. This motif binds ATO in vitro (Figure S5), and when it is mutated, the early phase of expression in Ch cells is abolished (Figure 4H). Conversely, this enhancer is ectopically activated when *ato* is misexpressed in the ectoderm (Figure 4I,J), but this ectopic activation is abolished when the E box motif is mutated (unpublished data). In contrast to direct activation by *ato*, the ES enhancer is active only after *sc* expression is switched off (*RfxB*: Figure 4K,L), suggesting that *sc* only indirectly activates *Rfx* in ES development. However, we note that the ES enhancer does contain two motifs conforming to the known SC binding site (GCAGSTG) and so it is possible that SC directly primes the *Rfx* gene for later expression in ES lineages. Overall, the evidence suggests that *Rfx* is a direct target of *ato* but not of *sc*, supporting the hypothesis that differences in *Rfx* regulation may be one means by which proneural factors regulate neuronal subtype characteristics.

Interestingly, the *ato*-related bHLH gene, *cato*, has a Ch-enriched expression pattern like *Rfx* [26]. Enhancer analysis revealed that *cato* too has separable Ch and ES enhancers [27]. The former contains an E_ATO_ site that is required for Ch expression, and it is ectopically activated upon misexpression of *ato*. Mutant analysis of *cato* reveals roles in cell cycle control and SOP fate maintenance but not in terminal differentiation [27]. Nevertheless, the similar regulation of *Rfx* and *cato* suggests that differential regulation of shared intermediate regulatory genes in different neuronal subtype lineages may be a common theme underlying subtype specification by *ato* and *sc*.

### The Forkhead Factor Gene, *fd3F*, Is a Ch-Specific Regulator of Differentiation

**Figure 5:**
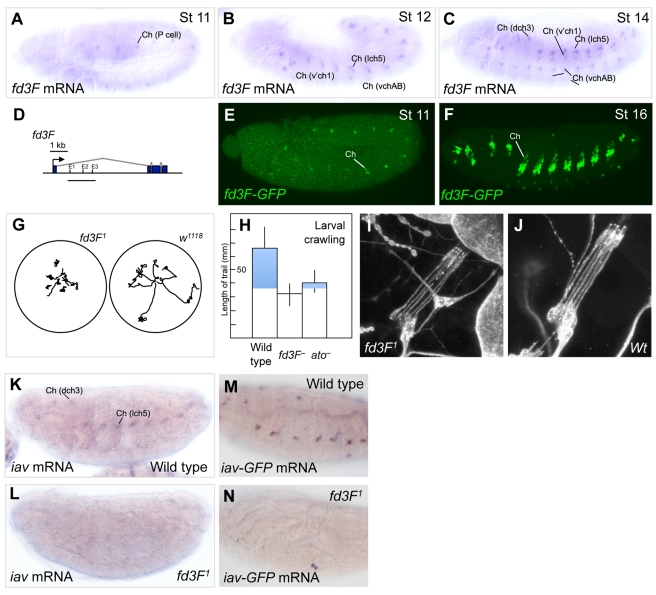
*fd3F* is downstream of *ato* function and required for Ch neuron function. (A–C) *fd3F* is expressed exclusively in Ch lineages from the precursor stage to differentiation. lch5, dch3, v'ch1, and vchAB are designations of specific Ch neurons or neuron groups [54]. (D) Schematic of *fd3F* gene, showing the location of the fragment tested for enhancer activity (E,F). The *fd3F* enhancer fragment drives GFP in Ch lineages. (E) Expression at stage 11 in Ch precursors. (F) Expression at stage 16 exclusively in Ch lineages. (G) Traces of larval movement for 2 min after being placed in middle of Petri dish. (H) Chart of larval locomotion test (as in (G)) of wildtype, *fd3F* ^−^, and *ato* ^−^ larvae. Locomotion is significantly reduced in *fd3F* ^−^ and *ato* ^−^ compared to wildtype (by *t* test, *p* = 1×10^−6^ and 5.7×10^−6^, respectively), consistent with defective Ch neurons. (I,J) Cluster of five Ch neurons in one abdominal segment of *fd3F* ^−^ (I) and wildtype (J) larva as revealed by anti-HRP staining. Ch neurons are grossly normal in the mutant. (K,L) Expression of *iav* is reduced in *fd3F* mutant embryo (L) compared to wild type (K). (M,N) Similarly, expression of an *iav*-GFP reporter gene construct (FGN, unpublished) is missing in *fd3F* mutant embryo (M) compared to wild type (N).

**Figure 6:**
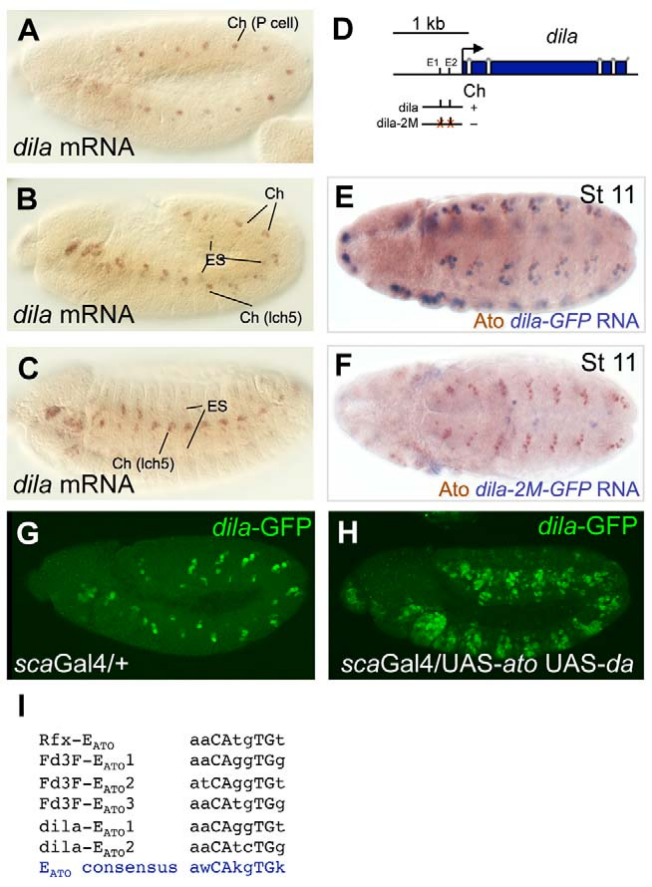
*dila* is a direct target of *ato* in the pathway to differentiation. (A–C) Expression of *dila* mRNA at stages 11, 12, and 15. *dila* is a Ch-enriched gene, being expressed strongly in Ch cells and weakly in ES cells. (D) Schematic of the first four exons of the *dila* gene, showing the location of the enhancer fragments tested, and the two E boxes within it. (E) *dila*-GFP in stage 11 embryo. GFP is driven by *dila* enhancer in early Ch cells, which express *ato*. (F) *dila*-2M-GFP. Mutation of two E_ATO_ boxes in the *dila* enhancer abolishes early Ch cell expression. (G,H) *dila*-GFP responds to *ato* misexpression. (G) Expression of *dila*-GFP in *sca*Gal4 driver background (wild type). (H) Ectopic expression of *dila*-GFP in embryo in which *ato* and its partner, *da*, are jointly misexpressed in the ectoderm. (I) Summary of E box motifs in potential *ato* target enhancers relative to the *ato*-specific consensus, E_ATO_ [7]. Note that *dila*-E_ATO_2 does not completely match the consensus and appears to bind ATO/DA more weakly in vitro (Figure S5).

The gene for the predicted Forkhead family transcription factor, *fd3F* (*CG12632*), is highly enriched in atoGFP cells (19.7-fold at t3; ranked 3^rd^). In contrast to *Rfx*, *fd3F* is expressed exclusively in Ch neurons from the precursor stage through to differentiation (Figure 5A–C), suggesting a specific role in Ch neuron specialisation. Its highly specific Ch expression pattern suggests that *fd3F* may be a direct target of *ato*. Reporter gene analysis identified an intronic Ch enhancer of *fd3F* that contains three *ato*-type E box motifs (Figures 5D–F, 6I). However, reporter expression does not appear strongly altered when these sites are mutated (unpublished data), suggesting that regulation may occur via other E box motifs. At present, therefore, although *fd3F* is a target of *ato*, we cannot conclude whether regulation is direct or indirect.

To ascertain *fd3F*'s function, we generated a mutation by P-element imprecise excision (FGN, in prep.). Mutant larvae and adult flies exhibit locomotion defects similar to those manifested in *ato* mutants (Figure 5G,H; FGN and APJ, in prep.) [4],[28]. Given the expression pattern of *fd3F*, these defects can be attributed to defective Ch neurons, which are required for proprioceptive feedback during locomotion. In *ato* mutants, such defective behaviour results from loss of Ch neurons. Immunohistochemical analysis suggests, however, that Ch neurons are mostly specified normally in *fd3F* mutants and little gross structural defect was observed in the neurons (Figure 5I,J; FGN, in prep.). Consistent with this, preliminary analysis of gene expression suggests that most ciliogenesis genes tested are not affected in *fd3F* mutants (FGN and APJ, in prep.).

We hypothesized, therefore, that *fd3F* regulates specialised aspects of Ch neuronal or ciliary physiology. The transient receptor potential (TRP) family of Ca^2+^ channels are particularly associated with sensory functions in a range of ciliary contexts [29]. In *Drosophila*, *nan* and *iav* encode subunits of a TRPV channel that are uniquely expressed in Ch neurons [17],[18]. The proteins are located in the Ch ciliary dendrite, where they are required for sensory transduction. We find that the expression of both *nan* and *iav* is strongly reduced in *fd3F* mutant embryos (Figure 5K–N, and unpublished data). Failure in regulation of *nan* and *iav* can therefore account for the defective Ch neuron function of *fd3F* mutants. In conclusion, *ato* directly or indirectly activates a transcriptional regulator concerned with Ch neuron physiology (specifically, Ch ciliary dendrite physiological specialisation).

### Ato Directly Regulates *dilatory*, a Gene Directly Involved in Differentiation

Whilst many early expressed differentiation genes are known or predicted *Rfx* targets, not all Ch-specific or Ch-enriched genes (nor ciliogenesis genes) have nearby X box motifs, suggesting that other intermediate regulatory factors remain to be discovered. Another possibility is that some early expressed differentiation genes may be directly regulated by proneural factors. Such genes include *CG1625* and *unc*, whose expression depends strongly on *ato* function (Table S9). Our analysis (LM and APJ, in prep.) shows that *CG1625*, which we name *dilatory* (*dila*), encodes a coiled-coil protein that localises to the basal body, and *dila* mutants exhibit defects in ciliary axonemal assembly. Together, these suggest that *dila* is a not a transcriptional regulator, but instead has a direct function in ciliary dendrite formation. Here, we examined the regulation of *dila*. The gene is highly expressed in early Ch cells (11-fold enriched at t1; ranked 10^th^), and *dila* RNA exhibits a Ch-enriched gene expression pattern in embryos (Figure 6A–C). However, it has no X box motif within 2 kb of its transcription start site. Its early expression raises the possibility that *dila* is directly regulated by *ato*. In vivo reporter gene analysis led to the identification of an enhancer required for *dila* expression in Ch cells (Figure 6D,E). Conversely, the reporter gene is misexpressed when *ato* is ectopically activated in the ectoderm (Figure 6G,H). This enhancer contains two sequences resembling E_ATO_ motifs, both of which bind ATO/DA in vitro (Figures 6I, S5). Mutation of these two motifs within this enhancer results in loss of early expression in Ch SOPs (Figure 6D,F) and loss of misexpression in response to ectopically activated *ato* (unpublished data). These data are consistent with direct regulation of *dila* by *ato* via one or both of these E_ATO_ motifs. We note that in a recent study of potential *ato* target genes in retinal development, similar evidence was presented to suggest that *dila* (as *CG1625*) is regulated by *ato* via these two motifs [30]. In conclusion, *dila* represents a differentiation gene that is directly controlled by a proneural factor, despite the gap between proneural factor expression and terminal differentiation.

## Discussion

**Figure 7:**
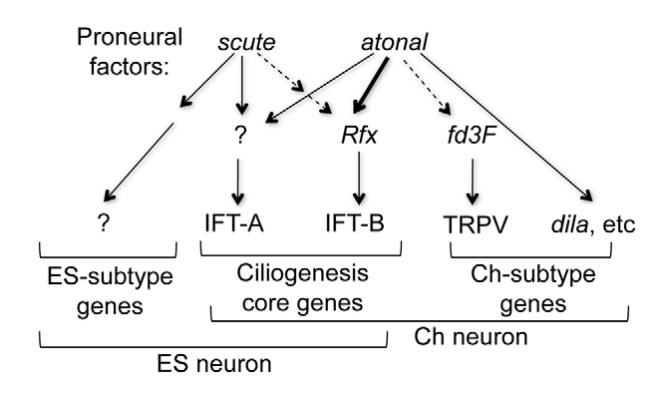
Summary of proposed regulatory interactions. Summary of proposed interactions leading from proneural genes to neuronal subtype differentiation. Solid and dashed arrows represent putative direct and indirect regulation, respectively. *ato* and *ac* regulate shared and unique aspects of sensory neuron differentiation. Ch ciliary specialisation is regulated by *ato* via several routes. (1) *ato* regulates a Ch-specific intermediate transcriptional regulatory (*fd3F*) that in turn regulates specialised aspects of sensory ciliary function. (2) *ato* regulates at least one differentiation gene directly (*dila*). (3) *Rfx* regulates a subset of ciliogenesis genes (including IFT-B genes) shared between sensory lineages, but differences in *Rfx* regulation by proneural genes *ato* and *sc* (only *ato* regulates *Rfx* directly) modulate these aspects. (4) The regulation of other aspects of differentiation and ciliogenesis (including IFT-A genes) does not depend on *Rfx*, suggesting further intermediate regulators remain to be discovered.

Numerous genetic and misexpression analyses in a range of organisms have shown that proneural factors influence a neuron's ultimate phenotype (including its subtype identity) at an early stage in its development [1]. However, the nature of this influence on the cell biological processes of neuronal differentiation has remained obscure. This study bridges the gap between early specification by the proneural factor, *ato*, and the differentiation of Ch neurons. The current model in both *Drosophila* and vertebrates is that proneural factors activate two types of target gene during neural precursor specification: a common target set for shared neuronal properties and a unique target set for subtype-specific properties [31]. Our data suggest that such neuronal subtype differences are ultimately controlled by proneural factors in several ways: by the differential regulation of both specific and common intermediate transcription factors, which in turn regulate genes for aspects of neuronal structural and functional differentiation, and by direct regulation of potential differentiation genes (Figure 7).

The proneural factors *ato* and *sc* commit cells to similar but distinct neural precursor fates: Ch and ES neurons are evolutionarily related cell types with similar but distinct structural and physiological properties. Notably, both are characterised by the possession of specialised ciliary-based dendrites [10]–[12]. Thus, ciliogenesis is a key pathway that must ultimately be activated in sensory neurons subsequent to proneural factor function. However, there are important differences between the dendrites of Ch and ES neurons. Ch dendrites have a more prototypically organised axonemal structure and possess a characteristic ciliary dilation—a specialisation that separates the Ch ciliary dendrite into functionally distinct zones [32]. Moreover, there is evidence for an active ‘beat’ of Ch cilia during sensory transduction [33]. In general, ES dendrites appear reduced in structure: although a basal body and short axoneme are present, the tip of the dendrite consists of a ‘tubular body’ of irregularly packed microtubules [10]. Thus the basic ciliogenesis pathway must be modulated differently in Ch and ES differentiation, and ultimately this must reflect a difference in function between *ato* and *sc* proneural factors. The ciliogenic regulator *Rfx* is expressed and required for both ES and Ch lineages, but it is more strongly and more persistently expressed in Ch lineages (the Ch-enriched pattern). This modulation of *Rfx* expression is at least partly due to differences in its regulation by proneural factors, since it appears to be a direct target of *ato* but not *sc*. We hypothesise that differences in *Rfx* regulation by the proneural factors lead to differences in implementation of a core cilia biogenesis program, thereby directly linking early proneural factor function with key differences of neuronal morphology. Consistent with this idea, our data show that several known or predicted ciliogenesis genes also exhibit this Ch-enriched pattern, and some of these are predicted or known *Rfx* targets [20].

In this view, the subtype differences between Ch and ES neurons are partly produced by quantitative differences in timing or level of expression of a common differentiation process, which ultimately depends on a qualitative difference in *Rfx* regulation by the proneural factors. A possible example of this is *CG6129*. This gene is a predicted *Rfx* target gene and is expressed in a Ch-enriched pattern (Figure S3) [20]. The homologous mouse protein (Rootletin) localises to the ciliary rootlet and is required for its formation [34]. Thus Ch-enriched expression of *CG6129* explains the presence of the ciliary rootlet in Ch neurons but not ES neurons [11],[12]. One prediction of this hypothesis is that overexpression of *Rfx* in ES neurons will upregulate Ch-enriched genes, and this is borne out by preliminary experiments that show an increase in *CG6129* expression in ES neurons upon *Rfx* overexpression (*sca*Gal4/UAS-*Rfx* embryos; LM and APJ, unpublished data). It is notable that differences in IFT activity are proposed to underlie differences in ciliary morphology [35] while RFX class factors have been associated with regulating genes for IFT in a variety of organisms [13]. Our work suggests that variations in *Rfx* expression level and timing should be explored as a possible factor in cilium diversity.

*fd3F* fits the more conventional view of a proneural target gene that implements a subtype-specific program of differentiation [31]. It is expressed downstream of *ato* uniquely in Ch neurons and regulates genes required for functional specialisation of the Ch ciliary dendrite. It is likely that Forkhead factors regulate specialisation of ciliogenesis in other organisms. In *C. elegans*, *FKH-2* is expressed widely early in development but is also required specifically for ciliary specialisation of one type of sensory neuron [36]. *Foxj1* in mice, *Xenopus*, and zebrafish appears to be required for the motile cilia of the lung airway and embryonic node, but not for primary cilia [37]–[39]. It remains to be determined whether *fd3F* regulates the machinery for the active beat that occurs in Ch dendrites as part of sensory transduction [33]. Together, our studies of *Rfx* and *fd3F* extend the previously limited knowledge of the gene regulatory network underlying ciliogenesis [13] and provide insight into how the core program may be modified to produce the highly specialised and diverse morphologies that cilia adopt for different functions [36].

Previous to this study, little was known about how *ato/sc* proneural genes control the acquisition of Ch/ES subtype identity, except that regulation of the Cut homeodomain transcription factor is involved. Mutant and misexpression analyses show that *cut* is a fate selector switch for ES identity downstream of *sc* [19],[40], but nothing is known of its mode of action or targets. Whereas *Rfx* and *fd3F* functions are likely to be confined to neuronal morphology, *cut* affects the identity of support cells too [41]. As a fate switch in the entire lineage, it appears likely that *cut* is involved in high-level fate specification (like proneural genes) rather than regulating aspects of differentiation directly. However, it is also possible that *cut* may repress ciliogenesis genes in ES neurons, either directly or by repressing *Rfx* expression. It will be important to integrate *cut* into the Ch/ES gene regulatory network in the future.

In our temporal expression profiling data, there is a steady increase in the number of known or suspected differentiation genes expressed in developing Ch cells. Many more are not expressed until after our analysis ends. Ciliogenesis is a highly intricate cellular process requiring the coordination of perhaps hundreds of genes [13],[42] and differences in expression onset may indicate prerequisite steps in the process of differentiation and ciliogenesis. A surprising observation was the significant number of ciliogenesis and differentiation genes that are expressed even at the earliest profiling time point. This is unexpected, since the earliest time point is predicted to be not only before differentiation but also even before cell divisions have generated the neurons. We suggest that further analysis of expression timing may lead to insights into the cell biology of ciliogenesis. The early activation of differentiation genes may reflect the rapid pace of development in the *Drosophila* embryo. Thus, early expression of ciliogenesis genes may provide components that prime cells for rapid cilium assembly later once differentiation has been triggered. Along these lines, our findings mirror striking observations of retinal ganglion cells, whose rapid differentiation within 15 minutes of the exit from mitosis has been taken to imply that genes required in postmitotic cells must be transcribed before cell division [43],[44]. A more intriguing possibility is that early expression reflects an orderly time course for ciliogenesis that begins many hours before the final cell division. For example, *unc* is thought to be required for the conversion of the mitotic centriole to ciliogenic basal body [14], but we found that the mRNA and fusion protein are expressed even in SOPs, several cell divisions before terminal differentiation. Interestingly, in mammals newly replicated centrioles mature over two cell cycles [45]. It is conceivable that the sensory neuron basal body might similarly need time to mature.

Since *Rfx* and some ciliogenesis genes are expressed in SOPs, what prevents ciliogenesis from being activated in the non-neuronal support cells? One possibility would be an extension of model recently proposed for the generation of support cell differences, in which Notch signalling between daughter cells confines the function of genes to one branch of the lineage [23]. This would predict that ciliogenesis genes and/or *Rfx* are Notch target genes. Another possibility is that some of the gene products are asymmetrically segregated. Thirdly, ciliogenesis may not be triggered until one or more key gene products are produced in the neuronal cell.

As a corollary, it will be important to explore further the gene regulatory network underlying the temporal and cell-type differences in ciliogenesis genes. Some early expressed differentiation genes are known or predicted *Rfx* targets [20]. This gives a rationale for the early regulation of *Rfx* by *ato* in Ch lineages. However, in both *C. elegans* and *D. melanogaster*, *Rfx* regulates only a subset of ciliogenesis genes (notably, it does not regulate IFT-A genes) [20]. Further studies on *ato* target genes and the ciliogenesis regulatory network in sensory neurons will identify other important regulators (Figure 7). It remains to be determined how many differentiation genes are, like *dila*, direct targets of *ato*. Interestingly, vertebrate proneural factors are hypothesised to regulate directly the transition from cycling neural progenitor (or neural stem cell) to postmitotic differentiating neuron. Perhaps *ato* has retained some part of an ancestral proneural factor function in direct regulation of terminal differentiation despite the subsequent evolution of SOPs that must undergo several divisions before differentiating.

## Materials and Methods

### *ato*-GFP Reporter Fly Stock

In order to label *ato*-expressing cells, a 2.6-kb fragment upstream of the *ato* gene was used to drive GFP expression in transgenic *Drosophila* embryos. After amplification from genomic DNA (Table S10 for primers), this fragment was cloned into pHStinger [46]. The plasmid was used to make transgenic fly lines by microinjection. One viable line, *ato*GFP.7, with high expression levels and lacking detectable ectopic GFP expression, was chosen for embryo dissociation and cell sorting. For expression profiling of *ato* mutant cells, *ato*GFP.7 was introduced into the *ato^1^* mutant background (a presumed null [4]). To minimise genetic background differences, the *ato*GFP.7; *ato^1^* line was backcrossed four times to the original *ato*GFP.7 stock. The two lines are therefore predicted to be approximately 97% isogenic.

### Embryo Dissociation and Cell Sorting

In brief, dechorionated *ato*GFP embryos were dissociated in Shields and Sang (S2) medium (Sigma) with 5% fetal bovine serum (Gibco) in a Dounce homogeniser with a loose pestle. Cells were pelleted by centrifugation and resuspended in protease solution (90% trypsin-EDTA (Sigma) in phosphate buffered saline). Incubation in this solution for 7 min increased the proportion of viable single cells as judged by Trypan Blue exclusion. Cells were subsequently washed twice in S2 medium. Cell suspensions were separated using a DakoCytomation MoFlo MLS flow cytometer. In each run, 3×10^5^ *ato*GFP+ and 1×10^6^ *ato*GFP− cells were collected. Cells were sorted into Schneider medium on ice, then pelleted and homogenised in RNA extraction buffer, and then snap frozen in liquid nitrogen. In all experiments the cell suspension was kept on ice from the time of trypsin treatment until the RNA was extracted from the sorted cells. Quantitation of RNA was carried out using QuantiTect SYBR Green RT-PCR kit (Qiagen) and a MJ Research Opticon thermal cycler. rpL32 was used as a control.

### Microarray Data Processing and Analysis

Using standard techniques recommended by Affymetrix (http://www.affymetrix.com/support/technical/manual/expression_manual.affx), RNA from sorted *ato*GFP+ and *ato*GFP− cells was used to probe Affymetrix *Drosophila* 2.0 microarray chips in quadruplicate using independent samples. ∼0.5 µg of RNA was converted to cDNA and amplified as cRNA using the 2-cycle protocol, before being biotin labelled and fragmented. The hybridisations were conducted at the Sir Henry Wellcome Functional Genomics Facility, Glasgow, UK. Quality control and normalisation of microarray expression data was performed using the Bioconductor package AffyPLM [47] using the standard RMA method with quantile normalisation. Differentially expressed genes between *ato*GFP+ and *ato*GFP− samples were identified using the Bioconductor package limma [48]. Lists of Affymetrix probe-set accessions were extracted from the analysis with the cut-off at a 1% FDR [49]. Affymetrix probe-sets were mapped to genomic locations using the Ensembl database PerlAPI [50],[51] and only those probe-sets that were not promiscuous (not mapping to more than one gene) with ≥50% of their oligomers were considered reliable and used to retrieve stable accessions of ‘trusted genes’.

### Protein Domain Profiling

Protein domain annotations for Pfam, Prosite, Superfamily, and Smart databases were retrieved from Ensembl for all trusted genes in our analyses (Ensembl v53 March 2009, Flybase Release FB2008_10 Dmel Release 5.13, Nov. 2008). The resulting data were parsed into genomic frequency tables for each domain from each source. To determine whether any domains were over-represented in our gene lists, we applied a corrected Fisher exact test [52] to the relative domain frequencies between list and genome. All domains that were over-represented with *p*≤0.05 were taken forward for further analysis.

### Immunohistochemistry

Standard methods of whole embryo immunohistochemistry were used. Antibodies used were: anti-Ato 1∶2000 [4], MAb22C10 1∶100, MAb21A6 1∶500, anti-GFP 1∶500 (Molecular Probes), and anti-Pericentrin (1∶500, kindly provided by J. Raff). Secondary antibodies were from Molecular Probes. mRNA in situ hybridisation to whole embryos were by standard methods. Primers for antisense RNA probes used are given in Table S13. For double RNA/protein labelling, the in situ hybridisation was conducted first followed by protein detection. For wild-type embryos, we used the *w^1118^* stock. The fly stock for the *uncGFP* fusion gene/protein was kindly provided by Maurice Kernan.

### Promoter Fusions

Fragments were amplified from genomic DNA and cloned into pHStinger. Primers used are given in Table S13. Transformants were made by microinjection into syncytial blastoderm embryos. In general, at least two independent transformant lines were tested for each construct. For E box site directed mutagenesis, we used the Stratagene Quickchange 2 kit. In each case, CANNTG was altered to AANNTT.

### Gel Retardation Assay

In vitro DNA binding assays were performed exactly as previously described using bacterially expressed ATO and DA proteins [7]. DNA probes used are shown in Table S13.

### *fd3F* Mutant Analysis

A deletion allele, *fd3F^1^*, was isolated by imprecise excision after P element mobilisation in the line, P{EP}EP1198. This deletes the 3′ end of the transcription unit and appears to be an RNA and protein null (FGN, manuscript in preparation).

### Larval Crawling Analysis

Wandering third instar larvae were placed individually on the centre of a layer of 1% agarose in a Petri dish. Larval movement was traced over a period of 2 min. Path lengths were obtained from traces using NIH ImageJ. Larvae tested were from the stocks, *ato^1^*, *fd3F^1^*, and *w^1118^* (wild type).

### Data Availability

All microarray data from the experiments described are available from the NCBI's GEO database with accession number GSE21520.

## Supporting Information

Figure S1 **FACS analysis of cells dissociated from time collections of embryos.** Shown are the regions harvested for *ato*GFP+ and *ato*GFP− cell samples and the percentage of cells in each area. (A) *ato*GFP embryos. (B) Non-GFP-expressing wild type embryos (Oregon R). (C) Embryos expressing GFP ubiquitously (*ubi*GFP).
(0.42 MB TIF)

Figure S2 **Representation of genes containing selected protein domains.** Transcription factor domains (such as the homeodomain, T-box, zinc-finger) are well represented at all time points, whereas domains associated with differentiation increase with time. The TPR domain is strongly associated with genes involved in Golgi trafficking and IFT. All domain counts shown are significantly enriched (*p*≤0.05).
(0.17 MB TIF)

Figure S3 **mRNA in situ hybridisation patterns of Ch differentially expressed genes.** (A) Pan-neural genes—expressed in both PNS and CNS cells. (B) Pan-sensory genes—expressed in PNS cells only. (C) Ch-enriched genes—expressed initially in Ch precursors, then all sensory lineages (CH and ES), and finally often persisting in Ch neurons only. (D) Ch-specific—expressed exclusively in some or all Ch lineages in the sensory nervous system. (E) Head-only—expressed in *ato*-dependent cells in the head (BO = Bolwig's Organ, the larval photoreceptive organ). Note that these categories are not rigid and there is much subtle variation within each type.
(4.47 MB TIF)

Figure S4 **Resampling analysis shows that** ***ato*** **-correlated genes are highly enriched for nearby RFX binding motifs (X boxes) at each time point.** In each case, significantly enriched genes (≥2-fold, 1% FDR) were selected and their 1-kb upstream sequences analysed for X box sequence matches. To sample the background distribution of such matches, random gene lists of equal size to the enriched gene list were selected and analysed for X boxes in a similar way. The results are plotted as the number of genes with X boxes within the gene list against sampling frequency. In each case the background distribution conforms to normal distribution (fitted curve shown). The position of the enriched gene list is shown by a star and arrow, with the degree of X box over-representation compared to that expected by chance and its associated *p* value (based on *z* test).
(0.64 MB TIF)

Figure S5 **In vitro DNA-binding analysis of E_ATO_ motifs from** ***Rfx*** **and** ***dila*** **enhancers.** A gel retardation assay showing the binding of ATO/DA heterodimers to oligonucleotide probes containing E_ATO_ motifs from the *RfxA* enhancer (*Rfx*-E_ATO_1) and *dila* enhancer (*dila*-E_ATO_1 and *dila*-E_ATO_2). Arrow indicates the protein-DNA complexes and arrowhead indicates the free probes. Note that binding to *dila*-E_ATO_2 appears somewhat weaker, correlating with its divergence from the known E_ATO_ binding consensus (Figure 6I) [7].
(0.75 MB TIF)

Table S1 **Top 100** ***ato*** **-correlated genes at time point t1.** A list of genes ranked by fold change (FC) (i.e., ratio of expression in *ato*GFP cells versus the rest of the embryo) (1% FDR).
(0.19 MB DOC)

Table S2 **Top 100** ***ato*** **-correlated genes at time point t2.** A list of genes ranked by fold change (FC) (i.e., ratio of expression in *ato*GFP cells versus the rest of the embryo) (1% FDR).
(0.19 MB DOC)

Table S3 **Top 100** ***ato*** **-correlated genes at time point t3.** A list of genes ranked by fold change (FC) (i.e., ratio of expression in *ato*GFP cells versus the rest of the embryo) (1% FDR).
(0.18 MB DOC)

Table S4 **Functional gene annotation analysis of genes that are over-represented at t1 in** ***ato*** **GFP cells in wild type embryos.** Significance is quantified by the corrected Fisher exact statistic [52]. Only the 50 most significant terms are shown. ‘PNS related’ refers to GO terms that include genes already known to be associated with PNS development. This information was used to assess the overall representation of PNS-related GO terms (Table S7).
(0.09 MB DOC)

Table S5 **Functional gene annotation analysis of genes that are over-represented at t2 in** ***ato*** **GFP cells in wild type embryos.** Significance is quantified by the corrected Fisher exact statistic [52]. Only the 50 most significant terms are shown. ‘PNS related’ refers to GO terms that include genes already known to be associated with PNS development. This information was used to assess the overall representation of PNS-related GO terms (Table S7).
(0.09 MB DOC)

Table S6 **Functional gene annotation analysis of genes that are over-represented at t3 in** ***ato*** **GFP cells in wild type embryos.** Significance is quantified by the corrected Fisher exact statistic [52]. Only the 50 most significant terms are shown. ‘PNS related’ refers to GO terms that include genes already known to be associated with PNS development. This information was used to assess the overall representation of PNS-related GO terms (Table S7).
(0.09 MB DOC)

Table S7 **Over-representation of PNS-related GO terms in the enriched GO term lists (Tables S4, S5, S6).** In this table, the enrichment factor represents the enrichment in PNS-related GO terms relative to similar sized random lists of genes as generated by bootstrap analysis: PNS related GO terms associated with random gene lists were retrieved. This process was repeated to produce a score distribution that approximates to a normal distribution according to the central limit theorem. The resulting distributions were normalised and a single location *z* test performed against the real PNS related GO term counts for the reference differentially expressed gene list. Enrichments were calculated against the random sample means.
(0.04 MB DOC)

Table S8 **Over-represented protein domains for combined data from t1–t3.** Shown are Pfam domains that are significantly over-represented among genes at any of the three time points (*p*<0.05 for enrichment in a particular time point), along with the genes in each family. Based on 1.5-fold over-expressed genes, 1% FDR.
(0.05 MB DOC)

Table S9 ***ato*** **-correlated genes at t3 that have been associated with cilia and/or basal body formation or function and/or are associated with an X box motif.** Genes are sorted by overall rank fold-enrichment in *ato*GFP cells versus the rest of the embryo (>1.5-fold enriched; 1% FDR).
(0.20 MB DOC)

Table S10 **Expression patterns of** ***ato*** **-correlated genes.** A summary of patterns observed from in situ hybridization carried out for this study.
(0.10 MB DOC)

Table S11 **Genes differentially expressed at t1 in** ***ato*** **GFP cells from wild-type but not in** ***ato*** **mutant embryos.** A table of genes that meet the following criteria: ≥2-fold differentially expressed in *ato*GFP cells from wild-type embryos (fc = ratio of expression in *ato*GFP cells versus the rest of the embryo) and <2-fold differentially expressed in *ato*GFP cells from *ato*-mutant embryos (versus the rest of the embryo) (1% FDR).
(0.11 MB DOC)

Table S12 **Potential** ***ato*** **target genes based on genes differentially represented in wild-type versus** ***ato*** **-mutant cells.** This table shows a subset of the genes in Table S11, selected based on the following additional criterion: ≥2-fold ratio between wild-type and mutant fold-change values (Wt/mut). Compared with the genes in Table S11, this list removes many genes that do not show a robust expression difference between wild type and mutant (i.e., for which differential expression is just above 2-fold in wild type embryos and just below 2-fold in mutant embryos). It is likely that many *ato* target genes are likely to be excluded by these stringent criteria, particularly those that are expressed widely in other parts of the nervous system or elsewhere in the embryo. A second factor that limits the number of potential targets identified in this way is that a proportion of Ch neurons still develop in ato mutant embryos due to redundancy with the closely related gene, *cato* [27].
(0.05 MB DOC)

Table S13 **Oligonucleotides used for generation of in situ hybridisation probes, GFP reporter constructs, and gel retardation assays.**
(0.08 MB DOC)

Text S1 **FACS isolation of** ***ato*** **GFP cells and validation.**
(0.04 MB DOC)

Text S2 **Functional gene annotation (GO analysis).**
(0.04 MB DOC)

Text S3 **Developmental progression in GO term over-representation.**
(0.03 MB DOC)

Text S4 **Analysis of** ***ato*** **-correlated genes for over-represented protein domains.**
(0.04 MB DOC)

Text S5 **Comparison with proneural cluster-expressed genes from a previous profiling analysis.**
(0.03 MB DOC)

## Abbreviations

Ch: chordotonal
dbd: dorsal bipolar dendritic neuron
DCBB: Drosophila cilia and basal body database
dmd: dorsal multidendritic neuron
ES: external sensory
FDR: false discovery rate
GFP: green fluorescent protein
GO: gene ontology
IFT: intraflagellar transport
TPR: tetratricopeptide repeat
