# Supplementary figures and images for: The Gene Regulatory Cascade Linking Proneural Specification with Differentiation in Drosophila Sensory Neurons
Source: PLoS Biol. 2011 Jan 4;9(1):e1000568. doi: 10.1371/journal.pbio.1000568 (PMC3023811; doi:10.1371/journal.pbio.1000568)

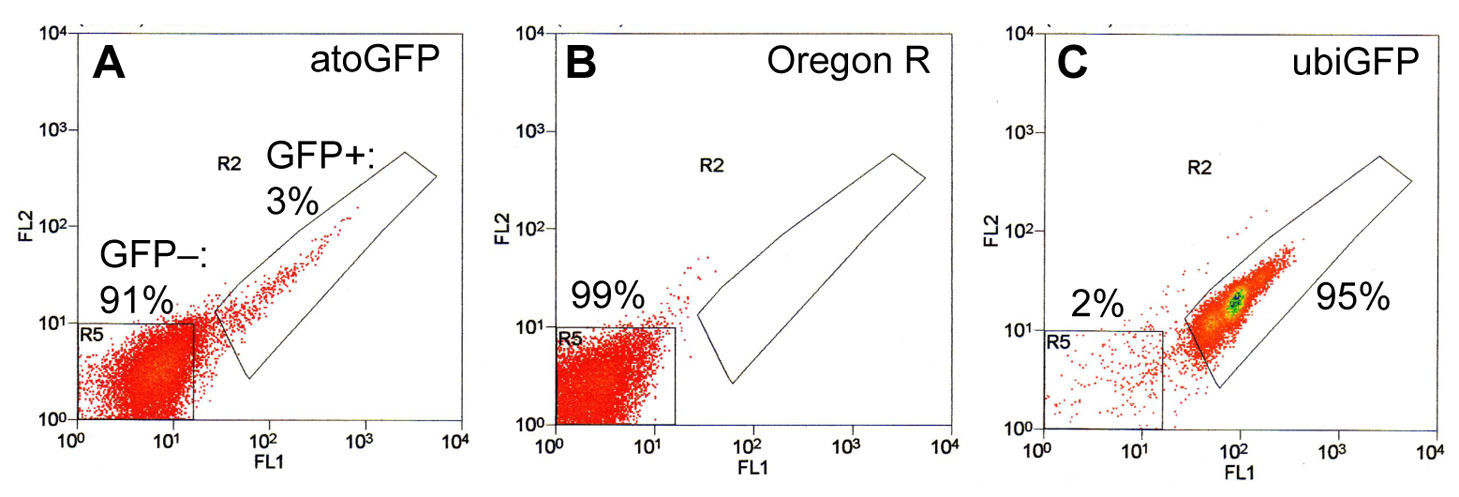

Supplement: Figure S1 — FACS analysis of cells dissociated from time collections of embryos. Shown are the regions harvested for atoGFP+ and atoGFP− cell samples and the percentage of cells in each area. (A) atoGFP embryos. (B) Non-GFP-expressing wild type embryos (Oregon R). (C) Embryos expressing GFP ubiquitously (ubiGFP). (0.42 MB TIF) [file pbio.1000568.s001.tif]

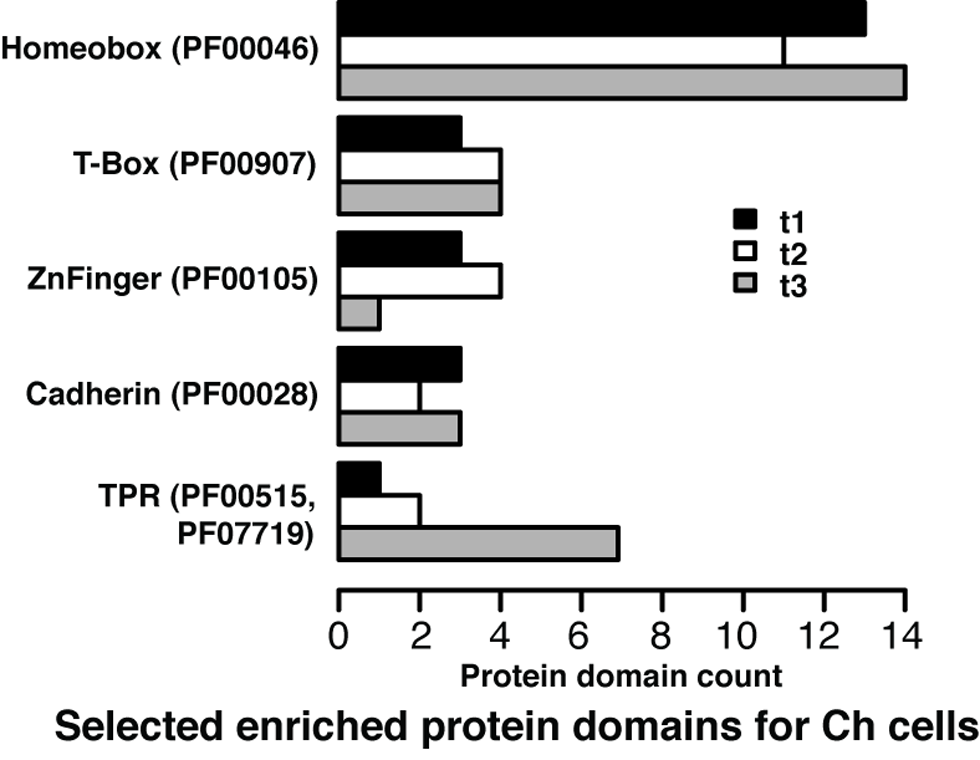

Supplement: Figure S2 — Representation of genes containing selected protein domains. Transcription factor domains (such as the homeodomain, T-box, zinc-finger) are well represented at all time points, whereas domains associated with differentiation increase with time. The TPR domain is strongly associated with genes involved in Golgi trafficking and IFT. All domain counts shown are significantly enriched (p≤0.05). (0.17 MB TIF) [file pbio.1000568.s002.tif]

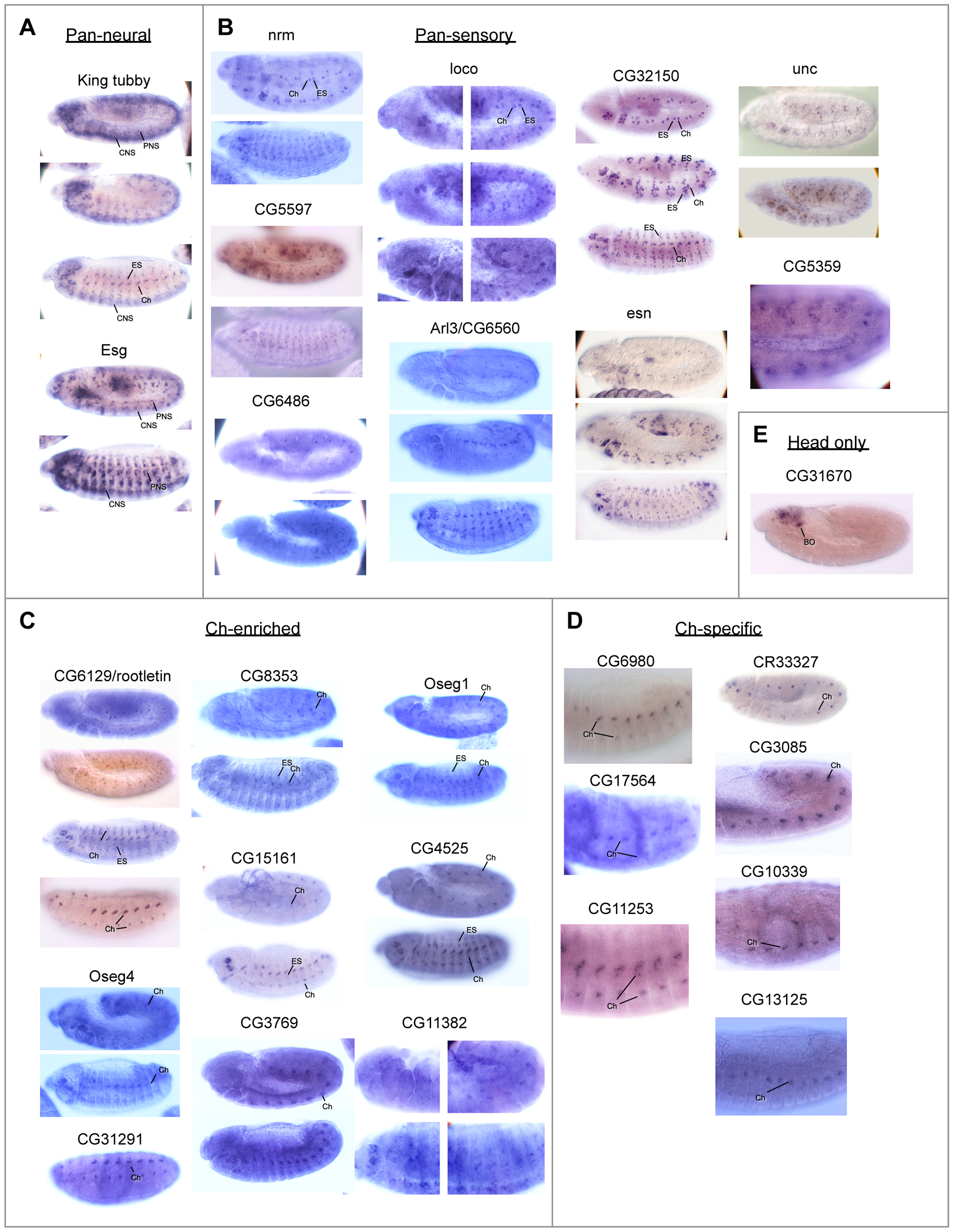

Supplement: Figure S3 — mRNA in situ hybridisation patterns of Ch differentially expressed genes. (A) Pan-neural genes—expressed in both PNS and CNS cells. (B) Pan-sensory genes—expressed in PNS cells only. (C) Ch-enriched genes—expressed initially in Ch precursors, then all sensory lineages (CH and ES), and finally often persisting in Ch neurons only. (D) Ch-specific—expressed exclusively in some or all Ch lineages in the sensory nervous system. (E) Head-only—expressed in ato-dependent cells in the head (BO = Bolwig's Organ, the larval photoreceptive organ). Note that these categories are not rigid and there is much subtle variation within each type. (4.47 MB TIF) [file pbio.1000568.s003.tif]

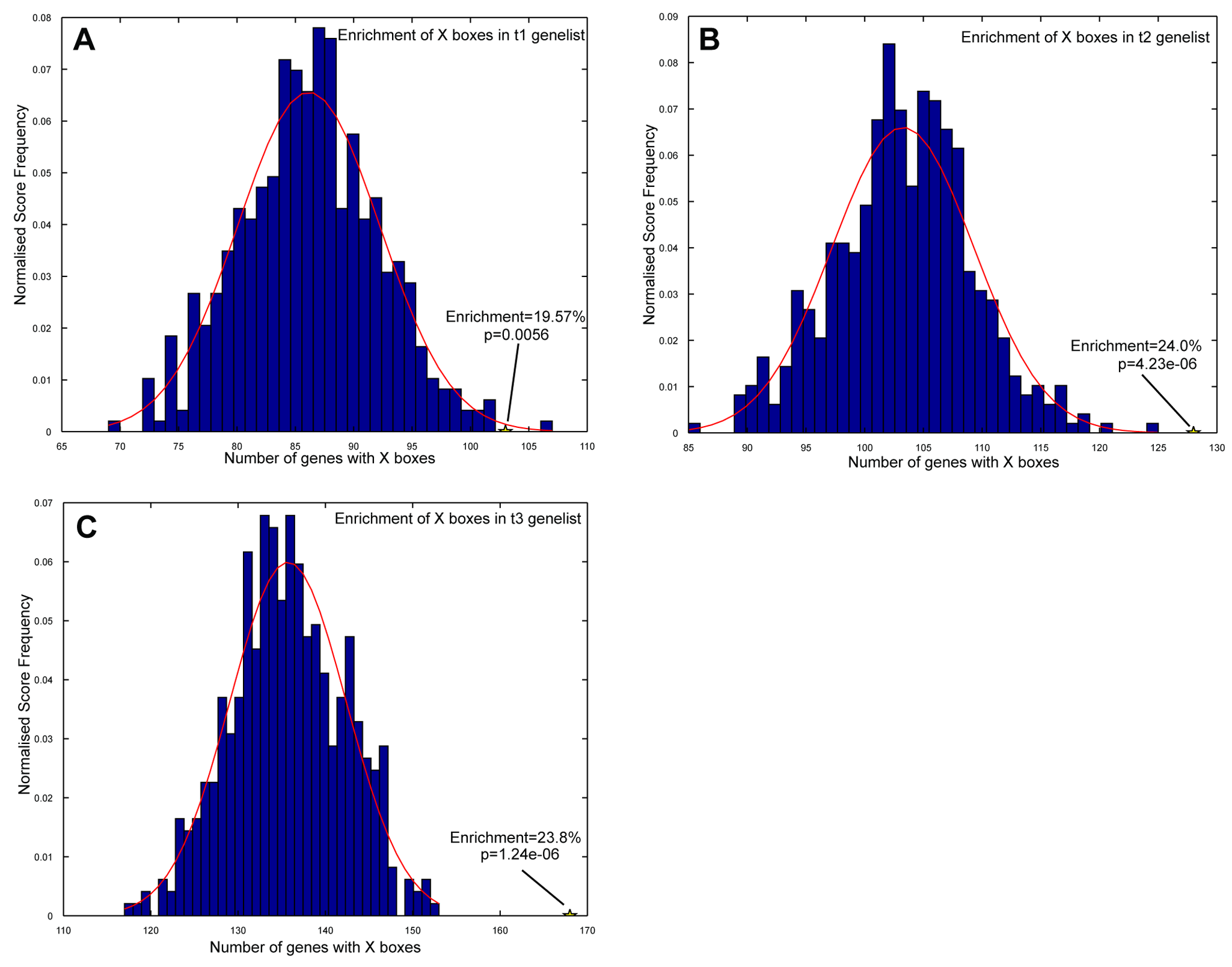

Supplement: Figure S4 — Resampling analysis shows that ato -correlated genes are highly enriched for nearby RFX binding motifs (X boxes) at each time point. In each case, significantly enriched genes (≥2-fold, 1% FDR) were selected and their 1-kb upstream sequences analysed for X box sequence matches. To sample the background distribution of such matches, random gene lists of equal size to the enriched gene list were selected and analysed for X boxes in a similar way. The results are plotted as the number of genes with X boxes within the gene list against sampling frequency. In each case the background distribution conforms to normal distribution (fitted curve shown). The position of the enriched gene list is shown by a star and arrow, with the degree of X box over-representation compared to that expected by chance and its associated p value (based on z test). (0.64 MB TIF) [file pbio.1000568.s004.tif]

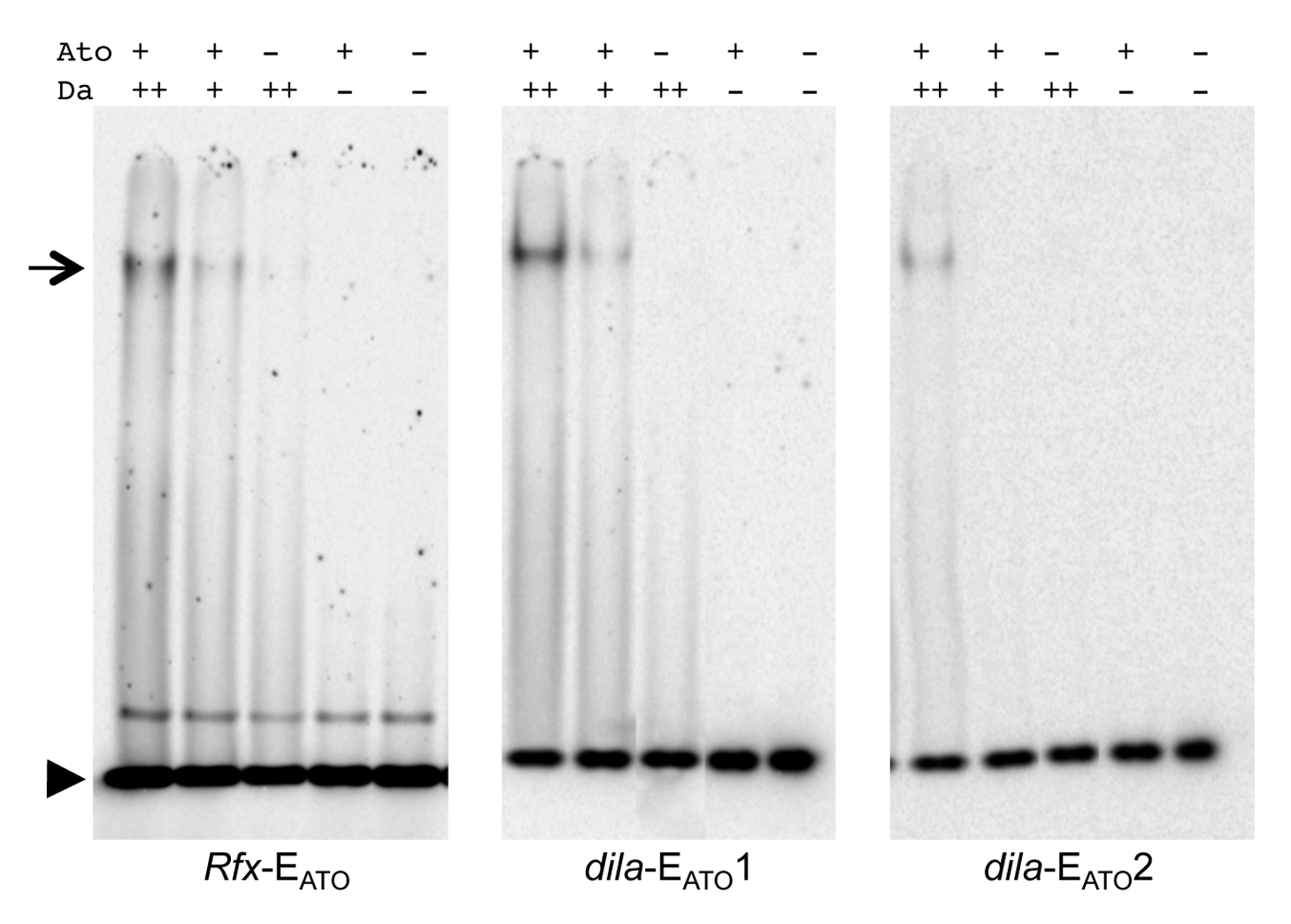

Supplement: Figure S5 — In vitro DNA-binding analysis of EATO motifs from Rfx and dila enhancers. A gel retardation assay showing the binding of ATO/DA heterodimers to oligonucleotide probes containing EATO motifs from the RfxA enhancer (Rfx-EATO1) and dila enhancer (dila-EATO1 and dila-EATO2). Arrow indicates the protein-DNA complexes and arrowhead indicates the free probes. Note that binding to dila-EATO2 appears somewhat weaker, correlating with its divergence from the known EATO binding consensus (Figure 6I) [7]. (0.75 MB TIF) [file pbio.1000568.s005.tif]
