# Supplementary material for: The Gene Regulatory Cascade Linking Proneural Specification with Differentiation in Drosophila Sensory Neurons
Source: PLoS Biol. 2011 Jan 4;9(1):e1000568. doi: 10.1371/journal.pbio.1000568 (PMC3023811; doi:10.1371/journal.pbio.1000568)
Supplement: Table S1 — Top 100 ato -correlated genes at time point t1. A list of genes ranked by fold change (FC) (i.e., ratio of expression in atoGFP cells versus the rest of the embryo) (1% FDR). (0.19 MB DOC) [file pbio.1000568.s006.doc]

**Table S1.** Top 100 *ato*-correlated genes at time point t1.

| **Rank** | **Gene symbol** | **Gene name** | **AffyDros2**  **probe-set id** | **Ensembl id** | **Flybase id** | **FC** |
| --- | --- | --- | --- | --- | --- | --- |
| 1 | *CG31670* | *CG31670* | 1624470_at | CG31670 | FBgn0031375 | 21.26 |
| 2 | *ss* | *spineless* | 1625198_at | CG6993 | FBgn0003513 | 21.08 |
| 3 | *toy* | *twin of eyeless* | 1633094_a_at | CG11186 | FBgn0019650 | 17.78 |
| 4 | *CG32150* | *CG32150* | 1640513_a_at | CG32150 | FBgn0052150 | 17.39 |
| 5 | *cato* | *cousin of atonal* | 1623462_at | CG7760 | FBgn0024249 | 16.95 |
| 6 | *cpo* | *couch potato* | 1624608_s_at | CG31243 | FBgn0000363 | 15.68 |
| 7 | *cpo* | *couch potato* | 1632644_s_at | CG31243 | FBgn0000363 | 15.38 |
| 8 | *dac* | *dachshund* | 1633341_s_at | CG4952 | FBgn0005677 | 12.12 |
| 9 | *ato* | *atonal* | 1640868_at | CG7508 | FBgn0010433 | 11.86 |
| 10 | *dila* | *dilatory* | 1623802_at | CG1625 | FBgn0033447 | 11.07 |
| 11 | *unc* | *uncoordinated* | 1637035_at | CG1501 | FBgn0003950 | 9.9 |
| 12 | *Rfx* | *Rfx* | 1628783_at | CG6312 | FBgn0020379 | 9.76 |
| 13 | *sens* | *senseless* | 1632294_at | CG32120 | FBgn0002573 | 9.52 |
| 14 | *B-H1* | *BarH1* | 1636203_at | CG5529 | FBgn0011758 | 8.46 |
| 15 | *CG17549* | *CG17549* | 1634507_s_at | CG17549 | FBgn0032774 | 8.31 |
| 16 | *CG32458* | *CG32458* | 1635083_at | CG32458 | FBgn0052458 | 8.02 |
| 17 | *ImpL3* | *Ecdysone-inducible gene L3* | 1635227_at | CG10160 | FBgn0001258 | 7.22 |
| 18 | *tll* | *tailless* | 1635993_at | CG1378 | FBgn0003720 | 6.9 |
| 19 | *Dll* | *Distal-less* | 1630237_a_at | CG3629 | FBgn0000157 | 6.84 |
| 20 | *esn* | *espinas* | 1632679_s_at | CG12833 | FBgn0028642 | 6.71 |
| 21 | *CG5597* | *CG5597* | 1638697_at | CG5597 | FBgn0034920 | 6.5 |
| 22 | *bi* | *bifid* | 1637049_at | CG3578 | FBgn0000179 | 6.04 |
| 23 | *CG9095* | *CG9095* | 1631280_at | CG9095 | FBgn0030617 | 5.71 |
| 24 | *fd3F* | *forkhead domain 3F* | 1639080_at | CG12632 | FBgn0061173 | 5.55 |
| 25 | *svp* | *seven up* | 1628779_a_at | CG11502 | FBgn0003651 | 5.54 |
| 26 | *sv* | *shaven* | 1636090_a_at | CG11049 | FBgn0005561 | 5.48 |
| 27 | *B-H2* | *BarH2* | 1640139_at | CG5488 | FBgn0004854 | 5.4 |
| 28 | *inv* | *invected* | 1623909_s_at | CG17835 | FBgn0001269 | 5 |
| 29 | *CG6959* | *CG6959* | 1633407_at | CG6959 | FBgn0037956 | 4.95 |
| 30 | *nrm* | *neuromusculin* | 1637057_at | CG8779 | FBgn0005629 | 4.84 |
| 31 | *Lim1* | *Lim1* | 1629733_at | CG11354 | FBgn0026411 | 4.69 |
| 32 | *CG30427* | *CG30427* | 1635524_at | CG30427 | FBgn0043792 | 4.4 |
| 33 | *CG15704* | *CG15704* | 1628430_at | CG15704 | FBgn0034103 | 4.35 |
| 34 | *Optix* | *Optix* | 1640296_a_at | CG18455 | FBgn0025360 | 4.32 |
| 35 | *nvy* | *nervy* | 1623977_at | CG3385 | FBgn0005636 | 4.22 |
| 36 | *CG32447* | *CG32447* | 1641477_at | CG32447 | FBgn0052447 | 4.21 |
| 37 | *gogo* | *golden goal* | 1625852_at | CG32227 | FBgn0052227 | 4.17 |
| 38 | *shd* | *shade* | 1638040_at | CG13478 | FBgn0003388 | 4.16 |
| 39 | *CG6129* | *CG6129* | 1634341_a_at | CG6129 | FBgn0039152 | 4.13 |
| 40 | *stan* | *starry night* | 1626087_at | CG11895 | FBgn0024836 | 3.86 |
| 41 | *disco-r* | *disco-related* | 1623846_at | CG32577 | FBgn0042650 | 3.8 |
| 42 | *cnc* | *cap-n-collar* | 1633379_s_at | CG17894 | FBgn0000338 | 3.78 |
| 43 | *Wsck* | *Wsck* | 1638192_a_at | CG31127 | FBgn0046685 | 3.77 |
| 44 | *disco* | *disconnected* | 1639940_at | CG9908 | FBgn0000459 | 3.73 |
| 45 | *CG11382* | *CG11382* | 1637360_at | CG11382 | FBgn0040367 | 3.7 |
| 46 | *spdo* | *sanpodo* | 1637254_at | CG31020 | FBgn0011716 | 3.66 |
| 47 | *al* | *aristaless* | 1639333_at | CG3935 | FBgn0000061 | 3.62 |
| 48 | *sad* | *shadow* | 1626485_at | CG14728 | FBgn0003312 | 3.57 |
| 49 | *CG8353* | *CG8353* | 1632345_at | CG8353 | FBgn0032002 | 3.56 |
| 50 | *Cad86C* | *Cad86C* | 1623112_at | CG4509 | FBgn0037840 | 3.56 |
| 51 | *scro* | *scarecrow* | 1635675_at | CG17594 | FBgn0028993 | 3.54 |
| 52 | *CG41452* | *CG41452* | 1628313_at | CG41452 | FBgn0084015 | 3.52 |
| 53 | *Atet* | *ABC transporter expressed in trachea* | 1629559_s_at | CG2969 | FBgn0020762 | 3.45 |
| 54 | *hh* | *hedgehog* | 1626527_at | CG4637 | FBgn0004644 | 3.4 |
| 55 | *CG13868* | *CG13868* | 1638183_at | CG13868 | FBgn0034501 | 3.36 |
| 56 | *CG32037* | *CG32037* | 1625275_at | CG32037 | FBgn0052037 | 3.23 |
| 57 | *Doc2* | *Dorsocross2* | 1628125_at | CG5187 | FBgn0035956 | 3.16 |
| 58 | *CG32150* | *CG32150* | 1627520_at | CG32150 | FBgn0052150 | 3.15 |
| 59 | *CG11069* | *CG11069* | 1631566_at | CG11069 | FBgn0039244 | 3.14 |
| 60 | *toy* | *twin of eyeless* | 1633512_at | CG11186 | FBgn0019650 | 3.12 |
| 61 | *CG31121* | *CG31121* | 1632118_s_at | CG31121 | FBgn0051121 | 3.11 |
| 62 | *w* | *white* | 1624393_at | CG2759 | FBgn0003996 | 3.11 |
| 63 | *CG17672* | *CG17672* | 1637708_a_at | CG17672 | FBgn0083978 | 3.06 |
| 64 | *CG17646* | *CG17646* | 1634296_s_at | CG17646 | FBgn0031362 | 3.04 |
| 65 | *CG17278* | *CG17278* | 1623565_at | CG17278 | FBgn0046763 | 3 |
| 66 | *halo* | *halo* | 1633058_at | CG7428 | FBgn0001174 | 2.99 |
| 67 | *CG6486* | *CG6486* | 1626642_at | CG6486 | FBgn0035922 | 2.95 |
| 68 | *CG12374* | *CG12374* | 1638361_at | CG12374 | FBgn0033774 | 2.92 |
| 69 | *en* | *engrailed* | 1627445_s_at | CG9015 | FBgn0000577 | 2.88 |
| 70 | *amd* | *alpha methyl dopa-resistant* | 1631281_a_at | CG10501 | FBgn0000075 | 2.88 |
| 71 | *Oseg4* | *Oseg4* | 1629688_at | CG2069 | FBgn0035264 | 2.79 |
| 72 | *CG30427* | *CG30427* | 1626793_at | CG30427 | FBgn0043792 | 2.75 |
| 73 | *vn* | *vein* | 1634520_at | CG10491 | FBgn0003984 | 2.75 |
| 74 | *CG16700* | *CG16700* | 1636835_at | CG16700 | FBgn0030816 | 2.74 |
| 75 | *CG13653* | *CG13653* | 1637824_at | CG13653 | FBgn0039288 | 2.72 |
| 76 | *Cyp12d1-p* | *Cyp12d1-p* | 1633401_s_at | CG30489 | FBgn0050489 | 2.72 |
| 77 | *Cyp12d1-d* | *Cyp12d1-d* | 1633401_s_at | CG33503 | FBgn0053503 | 2.72 |
| 78 | *CG11275* | *CG11275* | 1637513_at | CG11275 | FBgn0034706 | 2.64 |
| 79 | *sca* | *scabrous* | 1636998_at | CG17579 | FBgn0003326 | 2.63 |
| 80 | *sca* | *scabrous* | 1633936_a_at | CG17579 | FBgn0003326 | 2.62 |
| 81 | *CG6569* | *CG6569* | 1639974_a_at | CG6569 | FBgn0038909 | 2.53 |
| 82 | *CG33182* | *CG33182* | 1629788_at | CG33182 | FBgn0053182 | 2.53 |
| 83 | *tok* | *tolkin* | 1641053_s_at | CG6863 | FBgn0004885 | 2.51 |
| 84 | *for* | *foraging* | 1629886_s_at | CG10033 | FBgn0000721 | 2.51 |
| 85 | *svp* | *seven up* | 1635192_at | CG11502 | FBgn0003651 | 2.5 |
| 86 | *klu* | *klumpfuss* | 1629347_at | CG12296 | FBgn0013469 | 2.49 |
| 87 | *CG6330* | *CG6330* | 1640363_a_at | CG6330 | FBgn0039464 | 2.47 |
| 88 | *CG31464* | *CG31464* | 1639062_at | CG31464 | FBgn0051464 | 2.46 |
| 89 | *CG14223* | *CG14223* | 1625870_at | CG14223 | FBgn0031053 | 2.44 |
| 90 | *elB* | *elbow B* | 1631207_at | CG4220 | FBgn0004858 | 2.43 |
| 91 | *Toll-6* | *Toll-6* | 1624790_at | CG7250 | FBgn0036494 | 2.43 |
| 92 | *CG7047* | *CG7047* | 1641167_s_at | CG7047 | FBgn0035103 | 2.4 |
| 93 | *CG6560* | *CG6560* | 1638592_at | CG6560 | FBgn0038916 | 2.37 |
| 94 | *msi* | *musashi* | 1638125_a_at | CG5099 | FBgn0011666 | 2.36 |
| 95 | *rho* | *rhomboid* | 1635462_at | CG1004 | FBgn0004635 | 2.36 |
| 96 | *abd-A* | *abdominal A* | 1636558_a_at | CG10325 | FBgn0000014 | 2.35 |
| 97 | *Doc3* | *Dorsocross3* | 1629459_at | CG5093 | FBgn0035954 | 2.33 |
| 98 | *phm* | *phantom* | 1627188_at | CG6578 | FBgn0004959 | 2.33 |
| 99 | *CG17544* | *CG17544* | 1623069_s_at | CG17544 | FBgn0032775 | 2.32 |
| 100 | *dpp* | *decapentaplegic* | 1630026_s_at | CG9885 | FBgn0000490 | 2.32 |
|  |  |  |  |  |  |  |
