# Supplementary material for: The Gene Regulatory Cascade Linking Proneural Specification with Differentiation in Drosophila Sensory Neurons
Source: PLoS Biol. 2011 Jan 4;9(1):e1000568. doi: 10.1371/journal.pbio.1000568 (PMC3023811; doi:10.1371/journal.pbio.1000568)
Supplement: Table S2 — Top 100 ato -correlated genes at time point t2. A list of genes ranked by fold change (FC) (i.e., ratio of expression in atoGFP cells versus the rest of the embryo) (1% FDR). (0.19 MB DOC) [file pbio.1000568.s007.doc]

**Table S2.** Top 100 *ato*-correlated genes at time point t2.

| **Rank** | **Gene symbol** | **Gene name** | **AffyDros2**  **probe-set id** | **Ensembl id** | **Flybase id** | **FC** |
| --- | --- | --- | --- | --- | --- | --- |
| 1 | *dila* | *dilatory* | 1623802_at | CG1625 | FBgn0033447 | 17.73 |
| 2 | *toy* | *twin of eyeless* | 1633094_a_at | CG11186 | FBgn0019650 | 17.66 |
| 3 | *cato* | *cousin of atonal* | 1623462_at | CG7760 | FBgn0024249 | 15.64 |
| 4 | *ato* | *atonal* | 1640868_at | CG7508 | FBgn0010433 | 13.12 |
| 5 | *ss* | *spineless* | 1625198_at | CG6993 | FBgn0003513 | 12.96 |
| 6 | *CG32150* | *CG32150* | 1640513_a_at | CG32150 | FBgn0052150 | 11.8 |
| 7 | *CG31670* | *CG31670* | 1624470_at | CG31670 | FBgn0031375 | 11.45 |
| 8 | *CG5597* | *CG5597* | 1638697_at | CG5597 | FBgn0034920 | 11.19 |
| 9 | *CG6486* | *CG6486* | 1626642_at | CG6486 | FBgn0035922 | 10.38 |
| 10 | *dac* | *dachshund* | 1633341_s_at | CG4952 | FBgn0005677 | 10.21 |
| 11 | *cpo* | *couch potato* | 1624608_s_at | CG31243 | FBgn0000363 | 9.85 |
| 12 | *CG9095* | *CG9095* | 1631280_at | CG9095 | FBgn0030617 | 9.47 |
| 13 | *ImpL3* | *Ecdysone-inducible gene L3* | 1635227_at | CG10160 | FBgn0001258 | 8.59 |
| 14 | *CG17549* | *CG17549* | 1634507_s_at | CG17549 | FBgn0032774 | 8.26 |
| 15 | *unc* | *uncoordinated* | 1637035_at | CG1501 | FBgn0003950 | 8.18 |
| 16 | *cpo* | *couch potato* | 1632644_s_at | CG31243 | FBgn0000363 | 8.12 |
| 17 | *Rfx* | *Rfx* | 1628783_at | CG6312 | FBgn0020379 | 8.1 |
| 18 | *sens* | *senseless* | 1632294_at | CG32120 | FBgn0002573 | 7.63 |
| 19 | *CG32458* | *CG32458* | 1635083_at | CG32458 | FBgn0052458 | 6.6 |
| 20 | *CG15704* | *CG15704* | 1628430_at | CG15704 | FBgn0034103 | 6.18 |
| 21 | *esn* | *espinas* | 1632679_s_at | CG12833 | FBgn0028642 | 6.01 |
| 22 | *CG6129* | *CG6129* | 1634341_a_at | CG6129 | FBgn0039152 | 5.58 |
| 23 | *scro* | *scarecrow* | 1635675_at | CG17594 | FBgn0028993 | 5.54 |
| 24 | *fd3F* | *forkhead domain 3F* | 1639080_at | CG12632 | FBgn0061173 | 5.44 |
| 25 | *bi* | *bifid* | 1637049_at | CG3578 | FBgn0000179 | 5.17 |
| 26 | *nrm* | *neuromusculin* | 1637057_at | CG8779 | FBgn0005629 | 5.08 |
| 27 | *tll* | *tailless* | 1635993_at | CG1378 | FBgn0003720 | 5.02 |
| 28 | *CG11275* | *CG11275* | 1637513_at | CG11275 | FBgn0034706 | 4.92 |
| 29 | *B-H1* | *BarH1* | 1636203_at | CG5529 | FBgn0011758 | 4.52 |
| 30 | *Dll* | *Distal-less* | 1630237_a_at | CG3629 | FBgn0000157 | 4.23 |
| 31 | *inv* | *invected* | 1623909_s_at | CG17835 | FBgn0001269 | 4.17 |
| 32 | *CG3769* | *CG3769* | 1634763_at | CG3769 | FBgn0032119 | 4.12 |
| 33 | *CG13868* | *CG13868* | 1638183_at | CG13868 | FBgn0034501 | 4.06 |
| 34 | *Wsck* | *Wsck* | 1638192_a_at | CG31127 | FBgn0046685 | 3.99 |
| 35 | *CG6560* | *CG6560* | 1638592_at | CG6560 | FBgn0038916 | 3.85 |
| 36 | *CG13856* | *CG13856* | 1636383_at | CG13856 | FBgn0038959 | 3.85 |
| 37 | *CG6980* | *CG6980* | 1640684_at | CG6980 | FBgn0039228 | 3.84 |
| 38 | *B-H2* | *BarH2* | 1640139_at | CG5488 | FBgn0004854 | 3.77 |
| 39 | *CG14905* | *CG14905* | 1631651_at | CG14905 | FBgn0038452 | 3.69 |
| 40 | *al* | *aristaless* | 1639333_at | CG3935 | FBgn0000061 | 3.69 |
| 41 | *CG8353* | *CG8353* | 1632345_at | CG8353 | FBgn0032002 | 3.58 |
| 42 | *sv* | *shaven* | 1636090_a_at | CG11049 | FBgn0005561 | 3.57 |
| 43 | *spdo* | *sanpodo* | 1637254_at | CG31020 | FBgn0011716 | 3.57 |
| 44 | *CG34182* | *CG34182* | 1629350_at | CG34182 | FBgn0085211 | 3.56 |
| 45 | *nvy* | *nervy* | 1623977_at | CG3385 | FBgn0005636 | 3.54 |
| 46 | *Doc2* | *Dorsocross2* | 1628125_at | CG5187 | FBgn0035956 | 3.39 |
| 47 | *CG17672* | *CG17672* | 1637708_a_at | CG17672 | FBgn0083978 | 3.39 |
| 48 | *CG13653* | *CG13653* | 1637824_at | CG13653 | FBgn0039288 | 3.37 |
| 49 | *phm* | *phantom* | 1627188_at | CG6578 | FBgn0004959 | 3.35 |
| 50 | *hh* | *hedgehog* | 1626527_at | CG4637 | FBgn0004644 | 3.32 |
| 51 | *CG32037* | *CG32037* | 1625275_at | CG32037 | FBgn0052037 | 3.32 |
| 52 | *shd* | *shade* | 1638040_at | CG13478 | FBgn0003388 | 3.29 |
| 53 | *knrl* | *knirps-like* | 1626059_at | CG4761 | FBgn0001323 | 3.21 |
| 54 | *Cad86C* | *Cad86C* | 1623112_at | CG4509 | FBgn0037840 | 3.21 |
| 55 | *gt* | *giant* | 1629953_at | CG7952 | FBgn0001150 | 3.2 |
| 56 | *CG12374* | *CG12374* | 1638361_at | CG12374 | FBgn0033774 | 3.09 |
| 57 | *Blimp-1* | *Blimp-1* | 1632541_at | CG5249 | FBgn0035625 | 3.03 |
| 58 | *sad* | *shadow* | 1626485_at | CG14728 | FBgn0003312 | 3.01 |
| 59 | *disco-r* | *disco-related* | 1623846_at | CG32577 | FBgn0042650 | 2.99 |
| 60 | *CG12496* | *CG12496* | 1624501_at | CG12496 | FBgn0040385 | 2.99 |
| 61 | *CG7296* | *CG7296* | 1624069_at | CG7296 | FBgn0032283 | 2.92 |
| 62 | *CG32150* | *CG32150* | 1627520_at | CG32150 | FBgn0052150 | 2.89 |
| 63 | *CG16700* | *CG16700* | 1636835_at | CG16700 | FBgn0030816 | 2.88 |
| 64 | *halo* | *halo* | 1633058_at | CG7428 | FBgn0001174 | 2.88 |
| 65 | *gogo* | *golden goal* | 1625852_at | CG32227 | FBgn0052227 | 2.84 |
| 66 | *CG11069* | *CG11069* | 1631566_at | CG11069 | FBgn0039244 | 2.83 |
| 67 | *CG31464* | *CG31464* | 1639062_at | CG31464 | FBgn0051464 | 2.82 |
| 68 | *Sodh-2* | *Sorbitol dehydrogenase-2* | 1634742_at | CG4649 | FBgn0022359 | 2.81 |
| 69 | *Oseg4* | *Oseg4* | 1629688_at | CG2069 | FBgn0035264 | 2.8 |
| 70 | *Lim1* | *Lim1* | 1629733_at | CG11354 | FBgn0026411 | 2.79 |
| 71 | *Optix* | *Optix* | 1640296_a_at | CG18455 | FBgn0025360 | 2.77 |
| 72 | *Doc3* | *Dorsocross3* | 1629459_at | CG5093 | FBgn0035954 | 2.77 |
| 73 | *CG41452* | *CG41452* | 1628313_at | CG41452 | FBgn0084015 | 2.72 |
| 74 | *CG13125* | *CG13125* | 1636760_a_at | CG13125 | FBgn0032163 | 2.71 |
| 75 | *l(2)05510* | *lethal (2) 05510* | 1638079_at | CG13432 | FBgn0028622 | 2.66 |
| 76 | *en* | *engrailed* | 1627445_s_at | CG9015 | FBgn0000577 | 2.65 |
| 77 | *CG6330* | *CG6330* | 1640363_a_at | CG6330 | FBgn0039464 | 2.64 |
| 78 | *cnc* | *cap-n-collar* | 1633379_s_at | CG17894 | FBgn0000338 | 2.64 |
| 79 | *CG15161* | *CG15161* | 1632286_at | CG15161 | FBgn0032692 | 2.63 |
| 80 | *vn* | *vein* | 1634520_at | CG10491 | FBgn0003984 | 2.63 |
| 81 | *dpp* | *decapentaplegic* | 1630026_s_at | CG9885 | FBgn0000490 | 2.63 |
| 82 | *disco* | *disconnected* | 1639940_at | CG9908 | FBgn0000459 | 2.63 |
| 83 | *klu* | *klumpfuss* | 1629347_at | CG12296 | FBgn0013469 | 2.6 |
| 84 | *sca* | *scabrous* | 1633936_a_at | CG17579 | FBgn0003326 | 2.6 |
| 85 | *dib* | *disembodied* | 1629265_at | CG12028 | FBgn0000449 | 2.58 |
| 86 | *Sulf1* | *Sulfated* | 1635007_at | CG6725 | FBgn0040271 | 2.57 |
| 87 | *Crg-1* | *Circadianly Regulated Gene* | 1624373_at | CG32788 | FBgn0021738 | 2.57 |
| 88 | *CR42205* |  | 1624373_at | CR42205 | FBgn0085832 | 2.57 |
| 89 | *CG32137* | *CG32137* | 1625087_a_at | CG32137 | FBgn0052137 | 2.55 |
| 90 | *pgant2* | *polypeptide GalNAc transferase 2* | 1639535_at | CG3254 | FBgn0031530 | 2.54 |
| 91 | *wg* | *wingless* | 1632868_a_at | CG4889 | FBgn0004009 | 2.53 |
| 92 | *sca* | *scabrous* | 1636998_at | CG17579 | FBgn0003326 | 2.52 |
| 93 | *CG30085* | *CG30085* | 1626755_at | CG30085 | FBgn0050085 | 2.52 |
| 94 | *Wnt2* | *Wnt oncogene analog 2* | 1637438_at | CG1916 | FBgn0004360 | 2.52 |
| 95 | *Cby* | *Chibby* | 1625135_at | CG13415 | FBgn0067317 | 2.49 |
| 96 | *CG12972* | *CG12972* | 1630696_at | CG12972 | FBgn0037076 | 2.48 |
| 97 | *ppk20* | *pickpocket 20* | 1641001_at | CG7577 | FBgn0039676 | 2.47 |
| 98 | *insv* | *insensitive* | 1624375_at | CG3227 | FBgn0031434 | 2.47 |
| 99 | *CG1806* | *CG1806* | 1631261_at | CG1806 | FBgn0030360 | 2.45 |
| 100 | *CG7047* | *CG7047* | 1641167_s_at | CG7047 | FBgn0035103 | 2.44 |
|  |  |  |  |  |  |  |
