# Supplementary material for: The Gene Regulatory Cascade Linking Proneural Specification with Differentiation in Drosophila Sensory Neurons
Source: PLoS Biol. 2011 Jan 4;9(1):e1000568. doi: 10.1371/journal.pbio.1000568 (PMC3023811; doi:10.1371/journal.pbio.1000568)
Supplement: Table S3 — Top 100 ato -correlated genes at time point t3. A list of genes ranked by fold change (FC) (i.e., ratio of expression in atoGFP cells versus the rest of the embryo) (1% FDR). (0.18 MB DOC) [file pbio.1000568.s008.doc]

**Table S3.** Top 100 *ato*-correlated genes at time point t3.

| **Rank** | **Gene symbol** | **Gene name** | **AffyDros2**  **probe-set id** | **Ensembl id** | **Flybase id** | **FC** |
| --- | --- | --- | --- | --- | --- | --- |
| 1 | *CG6980* | *CG6980* | 1640684_at | CG6980 | FBgn0039228 | 25.74 |
| 2 | *CG14905* | *CG14905* | 1631651_at | CG14905 | FBgn0038452 | 20.39 |
| 3 | *fd3F* | *forkhead domain 3F* | 1639080_at | CG12632 | FBgn0061173 | 19.72 |
| 4 | *CG11253* | *CG11253* | 1636602_at | CG11253 | FBgn0036338 | 18.41 |
| 5 | *tipE* | *temperature-induced paralytic E* | 1626200_s_at | CG1232 | FBgn0003710 | 18.22 |
| 6 | *CG34297* |  | 1637157_at | CG34297 | FBgn0085326 | 18.01 |
| 7 | *CG17564* | *CG17564* | 1637094_at | CG17564 | FBgn0032768 | 16.41 |
| 8 | *CG34182* |  | 1629350_at | CG34182 | FBgn0085211 | 15.92 |
| 9 | *tipE* | *temperature-induced paralytic E* | 1624098_s_at | CG1232 | FBgn0003710 | 12.31 |
| 10 | *CG18675* | *CG18675* | 1624098_s_at | CG18675 | FBgn0040696 | 12.31 |
| 11 | *CG17639* | *CG17639* | 1628094_at | CG17639 | FBgn0038029 | 11.23 |
| 12 | *CG4525* | *CG4525* | 1635131_at | CG4525 | FBgn0038358 | 10.5 |
| 13 | *CG3769* | *CG3769* | 1634763_at | CG3769 | FBgn0032119 | 9.78 |
| 14 | *tectonic* | *tectonic* | 1625411_at | CG9227 | FBgn0031783 | 9.72 |
| 15 | *Cad96Cb* | *Cad96Cb* | 1639044_at | CG13664 | FBgn0039294 | 9.6 |
| 16 | *CG13125* | *CG13125* | 1636760_a_at | CG13125 | FBgn0032163 | 9.35 |
| 17 | *CG3085* | *CG3085* | 1627537_at | CG3085 | FBgn0034816 | 9.26 |
| 18 | *Art7* | *Arginine methyltransferase 7* | 1627537_at | CG9882 | FBgn0034817 | 9.26 |
| 19 | *dila* | *dilatory* | 1623802_at | CG1625 | FBgn0033447 | 9.25 |
| 20 | *CG31291* | *CG31291* | 1624002_a_at | CG31291 | FBgn0051291 | 8.77 |
| 21 | *btv* | *beethoven* | 1638983_at | CG15148 | FBgn0023096 | 8.66 |
| 22 | *CG10064* | *CG10064* | 1631786_at | CG10064 | FBgn0035724 | 8.28 |
| 23 | *CG13889* | *CG13889* | 1641002_at | CG13889 | FBgn0035168 | 8.25 |
| 24 | *CG17669* | *CG17669* | 1623294_at | CG17669 | FBgn0034352 | 8.06 |
| 25 | *CG32703* | *CG32703* | 1636384_at | CG32703 | FBgn0052703 | 7.9 |
| 26 | *CG10339* | *CG10339* | 1637886_at | CG10339 | FBgn0034972 | 7.55 |
| 27 | *CG32006* | *CG32006* | 1625727_at | CG32006 | FBgn0052006 | 7.15 |
| 28 | *CG14394* | *CG14394* | 1633481_at | CG14394 | FBgn0038079 | 6.98 |
| 29 | *CG15161* | *CG15161* | 1632286_at | CG15161 | FBgn0032692 | 6.93 |
| 30 | *CG14367* | *CG14367* | 1638710_at | CG14367 | FBgn0038170 | 6.65 |
| 31 | *CG9611* | *CG9611* | 1638710_at | CG9611 | FBgn0028487 | 6.65 |
| 32 | *CG18631* | *CG18631* | 1640213_at | CG18631 | FBgn0034239 | 6.08 |
| 33 | *CG32150* | *CG32150* | 1640513_a_at | CG32150 | FBgn0052150 | 5.94 |
| 34 | *CG6327* | *CG6327* | 1623632_s_at | CG6327 | FBgn0036115 | 5.9 |
| 35 | *CG15701* | *CG15701* | 1639248_at | CG15701 | FBgn0034095 | 5.86 |
| 36 | *CG7768* | *CG7768* | 1639564_a_at | CG7768 | FBgn0036415 | 5.71 |
| 37 | *boss* | *bride of sevenless* | 1628369_at | CG8285 | FBgn0000206 | 5.68 |
| 38 | *CG13617* | *CG13617* | 1630908_at | CG13617 | FBgn0039201 | 5.52 |
| 39 | *CG6129* | *CG6129* | 1634341_a_at | CG6129 | FBgn0039152 | 5.47 |
| 40 | *CG30441* | *CG30441* | 1630985_at | CG30441 | FBgn0050441 | 5.26 |
| 41 | *CG10395* | *CG10395* | 1630985_at | CG10395 | FBgn0033019 | 5.26 |
| 42 | *CG13857* | *CG13857* | 1633887_at | CG13857 | FBgn0038958 | 5.09 |
| 43 | *Rfx* | *Rfx* | 1628783_at | CG6312 | FBgn0020379 | 4.97 |
| 44 | *ham* | *hamlet* | 1628089_at | CG31753 | FBgn0045852 | 4.88 |
| 45 | *CG17258* | *CG17258* | 1641706_at | CG17258 | FBgn0031496 | 4.82 |
| 46 | *nompA* | *no mechanoreceptor potential A* | 1641377_a_at | CG13207 | FBgn0016047 | 4.7 |
| 47 | *CG17549* | *CG17549* | 1634507_s_at | CG17549 | FBgn0032774 | 4.68 |
| 48 | *CG5597* | *CG5597* | 1638697_at | CG5597 | FBgn0034920 | 4.63 |
| 49 | *osm-6* | *osm-6* | 1640025_at | CG9595 | FBgn0031829 | 4.57 |
| 50 | *CG13622* | *CG13622* | 1623500_at | CG13622 | FBgn0039202 | 4.56 |
| 51 | *Oseg6* | *Oseg6* | 1628733_at | CG11237 | FBgn0034452 | 4.49 |
| 52 | *CG8214* | *CG8214* | 1630315_at | CG8214 | FBgn0034037 | 4.49 |
| 53 | *CG5142* | *CG5142* | 1632369_at | CG5142 | FBgn0032470 | 4.46 |
| 54 | *CAP* | *CAP* | 1633353_s_at | CG18408 | FBgn0033504 | 4.45 |
| 55 | *CG14692* | *CG14692* | 1631147_at | CG14692 | FBgn0037836 | 4.42 |
| 56 | *Phlpp* | *PH domain leucine-rich repeat protein phosphatase* | 1623550_at | CG10493 | FBgn0032749 | 4.37 |
| 57 | *esn* | *espinas* | 1632679_s_at | CG12833 | FBgn0028642 | 4.26 |
| 58 | *Crg-1* | *Circadianly Regulated Gene* | 1624373_at | CG32788 | FBgn0021738 | 4.24 |
| 59 | *CR42205* |  | 1624373_at | CR42205 | FBgn0085832 | 4.24 |
| 60 | *CG14164* | *CG14164* | 1627055_at | CG14164 | FBgn0036057 | 4.23 |
| 61 | *CG6709* | *CG6709* | 1627055_at | CG6709 | FBgn0036056 | 4.23 |
| 62 | *CG1126* | *CG1126* | 1627157_at | CG1126 | FBgn0037280 | 4.22 |
| 63 | *CG5964* | *CG5964* | 1635108_at | CG5964 | FBgn0036206 | 4.2 |
| 64 | *CG5048* | *CG5048* | 1629835_at | CG5048 | FBgn0036437 | 4.2 |
| 65 | *CG15730* | *CG15730* | 1625536_at | CG15730 | FBgn0030395 | 4.16 |
| 66 | *CG32458* | *CG32458* | 1635083_at | CG32458 | FBgn0052458 | 4.12 |
| 67 | *cpo* | *couch potato* | 1624608_s_at | CG31243 | FBgn0000363 | 4.12 |
| 68 | *CG6652* | *CG6652* | 1624197_a_at | CG6652 | FBgn0036687 | 4.11 |
| 69 | *Oseg5* | *Oseg5* | 1640383_at | CG9333 | FBgn0032891 | 4.11 |
| 70 | *CG6971* | *CG6971* | 1627798_at | CG6971 | FBgn0037962 | 4.05 |
| 71 | *CG14253* | *CG14253* | 1625563_s_at | CG14253 | FBgn0039467 | 4 |
| 72 | *osm-1* | *osm-1* | 1633181_at | CG13809 | FBgn0035317 | 3.98 |
| 73 | *dtr* | *defective transmitter release* | 1635400_at | CG31623 | FBgn0023090 | 3.92 |
| 74 | *CG16789* | *CG16789* | 1629421_at | CG16789 | FBgn0037712 | 3.92 |
| 75 | *Dll* | *Distal-less* | 1630237_a_at | CG3629 | FBgn0000157 | 3.92 |
| 76 | *Osi6* | *Osiris 6* | 1625382_at | CG1151 | FBgn0027527 | 3.91 |
| 77 | *CG30492* | *CG30492* | 1637188_s_at | CG30492 | FBgn0050492 | 3.81 |
| 78 | *CG3259* | *CG3259* | 1641499_at | CG3259 | FBgn0038221 | 3.81 |
| 79 | *alphaTub85E* | *alpha-Tubulin at 85E* | 1623910_at | CG9476 | FBgn0003886 | 3.79 |
| 80 | *CG32392* | *CG32392* | 1640789_a_at | CG32392 | FBgn0052392 | 3.71 |
| 81 | *CG5280* | *CG5280* | 1638851_at | CG5280 | FBgn0035952 | 3.67 |
| 82 | *ss* | *spineless* | 1625198_at | CG6993 | FBgn0003513 | 3.61 |
| 83 | *CG13856* | *CG13856* | 1636383_at | CG13856 | FBgn0038959 | 3.59 |
| 84 | *CG6560* | *CG6560* | 1638592_at | CG6560 | FBgn0038916 | 3.58 |
| 85 | *sr* | *stripe* | 1639223_a_at | CG7847 | FBgn0003499 | 3.51 |
| 86 | *Cht2* | *Chitinase 2* | 1637421_at | CG2054 | FBgn0022702 | 3.48 |
| 87 | *Doc2* | *Dorsocross2* | 1628125_at | CG5187 | FBgn0035956 | 3.47 |
| 88 | *Cpr49Ac* | *Cuticular protein 49Ac* | 1631701_a_at | CG8502 | FBgn0033725 | 3.39 |
| 89 | *ato* | *atonal* | 1640868_at | CG7508 | FBgn0010433 | 3.35 |
| 90 | *CG8292* | *CG8292* | 1636724_at | CG8292 | FBgn0032004 | 3.29 |
| 91 | *sv* | *shaven* | 1636090_a_at | CG11049 | FBgn0005561 | 3.26 |
| 92 | *Cad88C* | *Cad88C* | 1637488_at | CG3389 | FBgn0038247 | 3.24 |
| 93 | *CG10089* | *CG10089* | 1633800_s_at | CG10089 | FBgn0036369 | 3.22 |
| 94 | *Wnt2* | *Wnt oncogene analog 2* | 1637438_at | CG1916 | FBgn0004360 | 3.18 |
| 95 | *bi* | *bifid* | 1637049_at | CG3578 | FBgn0000179 | 3.17 |
| 96 | *nAcRbeta-96A* | *nicotinic Acetylcholine Receptor beta 96A* | 1635349_a_at | CG6798 | FBgn0004118 | 3.17 |
| 97 | *CG32235* | *CG32235* | 1640919_at | CG32235 | FBgn0047330 | 3.12 |
| 98 | *CG13203* | *CG13203* | 1628436_at | CG13203 | FBgn0033628 | 3.12 |
| 99 | *CG16984* | *CG16984* | 1637969_at | CG16984 | FBgn0062517 | 3.09 |
| 100 | *CG9541* | *CG9541* | 1629772_at | CG9541 | FBgn0032083 | 3.07 |
