# Supplementary material for: The Gene Regulatory Cascade Linking Proneural Specification with Differentiation in Drosophila Sensory Neurons
Source: PLoS Biol. 2011 Jan 4;9(1):e1000568. doi: 10.1371/journal.pbio.1000568 (PMC3023811; doi:10.1371/journal.pbio.1000568)
Supplement: Table S4 — Functional gene annotation analysis of genes that are over-represented at t1 in ato GFP cells in wild type embryos. Significance is quantified by the corrected Fisher exact statistic [52]. Only the 50 most significant terms are shown. ‘PNS related’ refers to GO terms that include genes already known to be associated with PNS development. This information was used to assess the overall representation of PNS-related GO terms (Table S7). (0.09 MB DOC) [file pbio.1000568.s009.doc]

**Table S4.** Functional gene annotation analysis of genes that are over-represented at t1 in *ato*GFP cells in wild type embryos.

| **GO term name** | **Accession** | **List**  **term frequency** | **Genome term frequency** | **Corrected**  **Fisher exact statistic** | **PNS**  **related** |
| --- | --- | --- | --- | --- | --- |
| regulation of transcription, DNA-dependent | GO:0006355 | 57 | 421 | 4.21E-018 | Y |
| sensory organ development | GO:0007423 | 19 | 78 | 1.27E-010 | Y |
| genital disc development | GO:0035215 | 9 | 16 | 1.19E-008 |  |
| dendrite morphogenesis | GO:0048813 | 23 | 150 | 1.24E-008 | Y |
| midgut development | GO:0007494 | 11 | 28 | 1.36E-008 | Y |
| regulation of transcription | GO:0045449 | 26 | 192 | 1.55E-008 | Y |
| imaginal disc-derived wing morphogenesis | GO:0007476 | 23 | 158 | 3.29E-008 | Y |
| homophilic cell adhesion | GO:0007156 | 13 | 46 | 3.30E-008 |  |
| compound eye development | GO:0048749 | 19 | 111 | 5.29E-008 | Y |
| open tracheal system development | GO:0007424 | 24 | 177 | 6.04E-008 | Y |
| regulation of tube size, open tracheal system | GO:0035151 | 8 | 15 | 1.83E-007 |  |
| peripheral nervous system development | GO:0007422 | 19 | 121 | 2.08E-007 | Y |
| leg disc proximal/distal pattern formation | GO:0007479 | 10 | 29 | 3.04E-007 | Y |
| imaginal disc-derived wing vein specification | GO:0007474 | 11 | 38 | 4.10E-007 | Y |
| wing disc anterior/posterior pattern formation | GO:0048100 | 8 | 20 | 2.30E-006 | Y |
| genital disc anterior/posterior pattern formation | GO:0035224 | 6 | 9 | 2.78E-006 |  |
| ectoderm development | GO:0007398 | 9 | 28 | 2.83E-006 | Y |
| establishment or maintenance of cell polarity | GO:0007163 | 10 | 37 | 3.22E-006 |  |
| epidermal growth factor receptor signaling pathway | GO:0007173 | 11 | 49 | 5.42E-006 | Y |
| determination of genital disc primordium | GO:0035225 | 6 | 10 | 6.07E-006 | Y |
| sensory organ precursor cell fate determination | GO:0016360 | 8 | 23 | 7.11E-006 | Y |
| septate junction assembly | GO:0019991 | 8 | 25 | 1.36E-005 |  |
| heart development | GO:0007507 | 13 | 78 | 1.51E-005 | Y |
| ommatidial rotation | GO:0016318 | 8 | 29 | 4.10E-005 | Y |
| DNA replication | GO:0006260 | 12 | 74 | 4.71E-005 |  |
| cell fate commitment | GO:0045165 | 7 | 21 | 4.81E-005 | Y |
| asymmetric cell division | GO:0008356 | 8 | 30 | 5.25E-005 | Y |
| antennal morphogenesis | GO:0048800 | 6 | 15 | 8.32E-005 | Y |
| negative regulation of transcription | GO:0016481 | 8 | 33 | 1.03E-004 | Y |
| establishment of ommatidial polarity | GO:0042067 | 9 | 44 | 1.12E-004 | Y |
| branched duct epithelial cell fate determination, open tracheal system | GO:0046845 | 6 | 16 | 1.21E-004 | Y |
| sensory cilium assembly | GO:0035058 | 5 | 10 | 1.69E-004 |  |
| regulation of tube architecture, open tracheal system | GO:0035152 | 5 | 10 | 1.69E-004 |  |
| DNA replication initiation | GO:0006270 | 6 | 17 | 1.71E-004 |  |
| imaginal disc-derived wing vein morphogenesis | GO:0008586 | 9 | 48 | 2.15E-004 | Y |
| spiracle morphogenesis, open tracheal system | GO:0035277 | 6 | 18 | 2.36E-004 | Y |
| eye-antennal disc morphogenesis | GO:0007455 | 7 | 27 | 2.39E-004 | Y |
| nervous system development | GO:0007399 | 13 | 105 | 3.00E-004 | Y |
| signal transduction | GO:0007165 | 17 | 172 | 3.71E-004 |  |
| Notch signaling pathway | GO:0007219 | 11 | 79 | 4.05E-004 | Y |
| genital disc sexually dimorphic development | GO:0035263 | 4 | 6 | 4.17E-004 |  |
| maintenance of epithelial integrity, open tracheal system | GO:0035160 | 5 | 12 | 4.18E-004 | Y |
| neuron development | GO:0048666 | 8 | 41 | 4.48E-004 | Y |
| compound eye photoreceptor cell differentiation | GO:0001751 | 7 | 32 | 6.57E-004 | Y |
| calcium-dependent cell-cell adhesion | GO:0016339 | 8 | 44 | 7.07E-004 |  |
| bristle morphogenesis | GO:0008407 | 9 | 57 | 7.36E-004 | Y |
| imaginal disc pattern formation | GO:0007447 | 5 | 14 | 8.57E-004 | Y |
| glial cell migration | GO:0008347 | 6 | 23 | 8.66E-004 | Y |
| axonogenesis | GO:0007409 | 9 | 60 | 1.05E-003 | Y |
| Bolwig's organ morphogenesis | GO:0001746 | 5 | 15 | 1.17E-003 | Y |
