# Supplementary material for: The Gene Regulatory Cascade Linking Proneural Specification with Differentiation in Drosophila Sensory Neurons
Source: PLoS Biol. 2011 Jan 4;9(1):e1000568. doi: 10.1371/journal.pbio.1000568 (PMC3023811; doi:10.1371/journal.pbio.1000568)
Supplement: Table S5 — Functional gene annotation analysis of genes that are over-represented at t2 in ato GFP cells in wild type embryos. Significance is quantified by the corrected Fisher exact statistic [52]. Only the 50 most significant terms are shown. ‘PNS related’ refers to GO terms that include genes already known to be associated with PNS development. This information was used to assess the overall representation of PNS-related GO terms (Table S7). (0.09 MB DOC) [file pbio.1000568.s010.doc]

**Table S5.** Functional gene annotation analysis of genes that are over-represented at t2 in *ato*GFP cells in wild type embryos.

| **GO term name** | **Accession** | **List**  **term frequency** | **Genome**  **term frequency** | **Corrected**  **Fisher exact statistic** | **PNS**  **related** |
| --- | --- | --- | --- | --- | --- |
| regulation of transcription, DNA-dependent | GO:0006355 | 57 | 421 | 8.90E-012 | Y |
| DNA replication | GO:0006260 | 20 | 74 | 1.54E-009 |  |
| regulation of tube size, open tracheal system | GO:0035151 | 10 | 15 | 2.70E-009 |  |
| DNA replication initiation | GO:0006270 | 10 | 17 | 1.41E-008 |  |
| dendrite morphogenesis | GO:0048813 | 27 | 150 | 1.69E-008 | Y |
| sensory organ development | GO:0007423 | 18 | 78 | 1.57E-007 | Y |
| genital disc development | GO:0035215 | 9 | 16 | 1.63E-007 |  |
| DNA-dependent DNA replication | GO:0006261 | 11 | 27 | 2.12E-007 |  |
| open tracheal system development | GO:0007424 | 27 | 177 | 5.27E-007 | Y |
| establishment or maintenance of cell polarity | GO:0007163 | 12 | 37 | 7.73E-007 |  |
| septate junction assembly | GO:0019991 | 10 | 25 | 1.13E-006 |  |
| homophilic cell adhesion | GO:0007156 | 13 | 46 | 1.24E-006 |  |
| midgut development | GO:0007494 | 10 | 28 | 3.53E-006 | Y |
| leg disc proximal/distal pattern formation | GO:0007479 | 10 | 29 | 4.98E-006 | Y |
| peripheral nervous system development | GO:0007422 | 20 | 121 | 6.38E-006 | Y |
| imaginal disc-derived wing vein specification | GO:0007474 | 11 | 38 | 8.48E-006 | Y |
| signal transduction | GO:0007165 | 24 | 172 | 1.18E-005 |  |
| genital disc anterior/posterior pattern formation | GO:0035224 | 6 | 9 | 1.45E-005 |  |
| compound eye development | GO:0048749 | 18 | 111 | 2.69E-005 | Y |
| imaginal disc-derived wing morphogenesis | GO:0007476 | 22 | 158 | 3.06E-005 | Y |
| antennal morphogenesis | GO:0048800 | 7 | 15 | 3.06E-005 | Y |
| determination of genital disc primordium | GO:0035225 | 6 | 10 | 3.12E-005 | Y |
| asymmetric cell division | GO:0008356 | 9 | 30 | 5.85E-005 | Y |
| heart development | GO:0007507 | 14 | 78 | 9.30E-005 | Y |
| regulation of transcription | GO:0045449 | 23 | 192 | 1.93E-004 | Y |
| calcium-dependent cell-cell adhesion | GO:0016339 | 10 | 44 | 2.09E-004 |  |
| wing disc anterior/posterior pattern formation | GO:0048100 | 7 | 20 | 2.24E-004 | Y |
| DNA repair | GO:0006281 | 12 | 64 | 2.34E-004 |  |
| cilium assembly | GO:0042384 | 10 | 45 | 2.51E-004 |  |
| ommatidial rotation | GO:0016318 | 8 | 29 | 3.35E-004 | Y |
| axon guidance | GO:0007411 | 23 | 203 | 4.27E-004 | Y |
| glial cell migration | GO:0008347 | 7 | 23 | 5.42E-004 | Y |
| sensory organ precursor cell fate determination | GO:0016360 | 7 | 23 | 5.42E-004 | Y |
| branched duct epithelial cell fate determination, open tracheal system | GO:0046845 | 6 | 16 | 5.80E-004 | Y |
| apical protein localization | GO:0045176 | 5 | 10 | 6.13E-004 |  |
| regulation of tube architecture, open tracheal system | GO:0035152 | 5 | 10 | 6.13E-004 |  |
| posterior head segmentation | GO:0035289 | 5 | 10 | 6.13E-004 |  |
| eggshell chorion gene amplification | GO:0007307 | 7 | 25 | 8.98E-004 |  |
| genital disc sexually dimorphic development | GO:0035263 | 4 | 6 | 1.12E-003 |  |
| imaginal disc growth | GO:0007446 | 7 | 26 | 1.13E-003 |  |
| eye-antennal disc morphogenesis | GO:0007455 | 7 | 27 | 1.41E-003 | Y |
| maintenance of epithelial integrity, open tracheal system | GO:0035160 | 5 | 12 | 1.48E-003 | Y |
| cell adhesion | GO:0007155 | 17 | 142 | 1.63E-003 | Y |
| ectoderm development | GO:0007398 | 7 | 28 | 1.74E-003 | Y |
| motor axon guidance | GO:0008045 | 7 | 29 | 2.12E-003 | Y |
| anterior head segmentation | GO:0035288 | 4 | 7 | 2.16E-003 |  |
| epidermal growth factor receptor signaling pathway | GO:0007173 | 9 | 49 | 2.24E-003 | Y |
| nerve maturation | GO:0021682 | 3 | 3 | 2.45E-003 |  |
| establishment of planar polarity | GO:0001736 | 9 | 50 | 2.57E-003 | Y |
| imaginal disc pattern formation | GO:0007447 | 5 | 14 | 2.97E-003 | Y |
| neuron development | GO:0048666 | 8 | 41 | 3.15E-003 | Y |
