# Supplementary material for: The Gene Regulatory Cascade Linking Proneural Specification with Differentiation in Drosophila Sensory Neurons
Source: PLoS Biol. 2011 Jan 4;9(1):e1000568. doi: 10.1371/journal.pbio.1000568 (PMC3023811; doi:10.1371/journal.pbio.1000568)
Supplement: Table S6 — Functional gene annotation analysis of genes that are over-represented at t3 in ato GFP cells in wild type embryos. Significance is quantified by the corrected Fisher exact statistic [52]. Only the 50 most significant terms are shown. ‘PNS related’ refers to GO terms that include genes already known to be associated with PNS development. This information was used to assess the overall representation of PNS-related GO terms (Table S7). (0.09 MB DOC) [file pbio.1000568.s011.doc]

**Table S6.** Functional gene annotation analysis of genes that are over-represented at t3 in *ato*GFP cells in wild type embryos.

| **GO term name** | **Accession** | **List**  **term frequency** | **Genome**  **term frequency** | **Corrected**  **Fisher exact statistic** | **PNS**  **related** |
| --- | --- | --- | --- | --- | --- |
| cilium assembly | GO:0042384 | 22 | 45 | 4.05E-023 |  |
| regulation of transcription, DNA-dependent | GO:0006355 | 43 | 421 | 1.33E-015 | Y |
| homophilic cell adhesion | GO:0007156 | 14 | 46 | 1.99E-011 |  |
| sensory cilium assembly | GO:0035058 | 8 | 10 | 1.31E-010 |  |
| calcium-dependent cell-cell adhesion | GO:0016339 | 12 | 44 | 3.06E-009 |  |
| leg disc proximal/distal pattern formation | GO:0007479 | 9 | 29 | 1.79E-007 | Y |
| regulation of tube size, open tracheal system | GO:0035151 | 7 | 15 | 4.19E-007 |  |
| genital disc development | GO:0035215 | 7 | 16 | 6.84E-007 |  |
| open tracheal system development | GO:0007424 | 18 | 177 | 8.79E-007 | Y |
| eye-antennal disc morphogenesis | GO:0007455 | 8 | 27 | 1.69E-006 | Y |
| regulation of transcription | GO:0045449 | 18 | 192 | 2.74E-006 | Y |
| wing disc anterior/posterior pattern formation | GO:0048100 | 7 | 20 | 3.43E-006 | Y |
| sensory perception of sound | GO:0007605 | 7 | 23 | 8.87E-006 | Y |
| antennal morphogenesis | GO:0048800 | 6 | 15 | 1.19E-005 | Y |
| septate junction assembly | GO:0019991 | 7 | 25 | 1.54E-005 |  |
| genital disc anterior/posterior pattern formation | GO:0035224 | 5 | 9 | 1.97E-005 |  |
| wing disc proximal/distal pattern formation | GO:0007473 | 4 | 6 | 1.25E-004 | Y |
| imaginal disc pattern formation | GO:0007447 | 5 | 14 | 1.83E-004 | Y |
| imaginal disc-derived wing vein specification | GO:0007474 | 7 | 38 | 2.05E-004 | Y |
| signal transduction | GO:0007165 | 14 | 172 | 2.06E-004 |  |
| anterior head segmentation | GO:0035288 | 4 | 7 | 2.46E-004 |  |
| pericardial cell differentiation | GO:0007513 | 4 | 8 | 4.24E-004 | Y |
| ommatidial rotation | GO:0016318 | 6 | 29 | 4.44E-004 | Y |
| heart development | GO:0007507 | 9 | 78 | 4.47E-004 | Y |
| sensory organ development | GO:0007423 | 9 | 78 | 4.47E-004 | Y |
| axonogenesis | GO:0007409 | 8 | 60 | 4.51E-004 | Y |
| spiracle morphogenesis, open tracheal system | GO:0035277 | 5 | 18 | 5.66E-004 | Y |
| dorsal/ventral pattern formation, imaginal disc | GO:0007450 | 4 | 9 | 6.66E-004 |  |
| compound eye photoreceptor cell differentiation | GO:0001751 | 6 | 32 | 7.24E-004 | Y |
| epidermal growth factor receptor signaling pathway | GO:0007173 | 7 | 49 | 8.72E-004 | Y |
| microtubule-based movement | GO:0007018 | 11 | 130 | 9.49E-004 |  |
| determination of genital disc primordium | GO:0035225 | 4 | 10 | 9.82E-004 | Y |
| posterior head segmentation | GO:0035289 | 4 | 10 | 9.82E-004 |  |
| branch fusion, open tracheal system | GO:0035147 | 5 | 21 | 1.09E-003 | Y |
| compound eye development | GO:0048749 | 10 | 111 | 1.16E-003 | Y |
| imaginal disc-derived wing morphogenesis | GO:0007476 | 12 | 158 | 1.24E-003 | Y |
| segment polarity determination | GO:0007367 | 7 | 53 | 1.34E-003 |  |
| establishment or maintenance of cell polarity | GO:0007163 | 6 | 37 | 1.46E-003 |  |
| labial disc development | GO:0035217 | 3 | 4 | 1.64E-003 |  |
| analia development | GO:0007487 | 3 | 4 | 1.64E-003 |  |
| optic lobe placode development | GO:0001748 | 3 | 4 | 1.64E-003 |  |
| ventral midline development | GO:0007418 | 3 | 4 | 1.64E-003 |  |
| eye-antennal disc development | GO:0035214 | 4 | 12 | 1.86E-003 |  |
| eye development | GO:0001654 | 4 | 12 | 1.86E-003 | Y |
| haltere development | GO:0007482 | 4 | 12 | 1.86E-003 | Y |
| wing disc morphogenesis | GO:0007472 | 4 | 13 | 2.44E-003 | Y |
| establishment of imaginal disc-derived wing hair orientation | GO:0001737 | 5 | 26 | 2.59E-003 | Y |
| dendrite morphogenesis | GO:0048813 | 11 | 150 | 2.79E-003 | Y |
| establishment of ommatidial polarity | GO:0042067 | 6 | 44 | 3.26E-003 | Y |
| ectoderm development | GO:0007398 | 5 | 28 | 3.47E-003 | Y |
