# Supplementary material for: The Gene Regulatory Cascade Linking Proneural Specification with Differentiation in Drosophila Sensory Neurons
Source: PLoS Biol. 2011 Jan 4;9(1):e1000568. doi: 10.1371/journal.pbio.1000568 (PMC3023811; doi:10.1371/journal.pbio.1000568)
Supplement: Table S7 — Over-representation of PNS-related GO terms in the enriched GO term lists (Tables S4, S5, S6). In this table, the enrichment factor represents the enrichment in PNS-related GO terms relative to similar sized random lists of genes as generated by bootstrap analysis: PNS related GO terms associated with random gene lists were retrieved. This process was repeated to produce a score distribution that approximates to a normal distribution according to the central limit theorem. The resulting distributions were normalised and a single location z test performed against the real PNS related GO term counts for the reference differentially expressed gene list. Enrichments were calculated against the random sample means. (0.04 MB DOC) [file pbio.1000568.s012.doc]

**Table S7.** Over-representation of PNS-related GO terms in the enriched GO term lists (Tables S4–6).

| Stage | Over-represented GO terms (total) | Over-represented PNS related GO terms | Enrichment factor over expected | Corrected Fisher exact statistic |
| --- | --- | --- | --- | --- |
| t1 | 141 | 79 | 2.94 | 1.0x10^-39^ |
| t2 | 142 | 70 | 2.58 | 4.5x10^-24^ |
| t3 | 114 | 71 | 3.26 | 3.9x10^-39^ |
