# Supplementary material for: The Gene Regulatory Cascade Linking Proneural Specification with Differentiation in Drosophila Sensory Neurons
Source: PLoS Biol. 2011 Jan 4;9(1):e1000568. doi: 10.1371/journal.pbio.1000568 (PMC3023811; doi:10.1371/journal.pbio.1000568)
Supplement: Table S8 — Over-represented protein domains for combined data from t1–t3. Shown are Pfam domains that are significantly over-represented among genes at any of the three time points (p<0.05 for enrichment in a particular time point), along with the genes in each family. Based on 1.5-fold over-expressed genes, 1% FDR. (0.05 MB DOC) [file pbio.1000568.s013.doc]

**Table S8.** Over-represented protein domains for combined data from t1–t3.

| **Domain name** | **Pfam accession** | **Count** | **Genes** |
| --- | --- | --- | --- |
| ABC transporter | PF00005  PF01061 | 6 | *w, Mdr49, Atet, CG17646, CG11069, CG31121* |
| Cadherin | PF00028 | 7 | *ds, stan, Cad74A, Cad86C, Cad88C, Cad96Cb, Cad99C* |
| Homeobox | PF00046 | 22 | *abd-A, Abd-B, al, Dll, en, inv, lab, prd, B-H2, B-H1, Awh, ara, caup, toy, dve, Optix, Lim1, scro, CG4136, toe, HGTX, vvl* |
| Cytochrome P450 | PF00067 | 6 | *dib, sad, shd, phm, Cyp18a1, Cyp301a1* |
| Zinc finger C4 type | PF00105 | 4 | *kni, knrl, svp, tll* |
| Paired box domain | PF00292 | 4 | *prd, sv, toy, toe* |
| Tetratricopeptide repeat (TPR) | PF00515  PF07719 | 7 | *nompB, BBS8, CG5142, BBS4, CG6915, CG6980, CG34297* |
| Leucine rich repeat (LRR) | PF00560 | 11 | *tilB, dtr, Tollo, CG13125, Phlpp, Als, CG11136, Toll-6, CG6959, CG12402, CG4221* |
| T box transcription factor | PF00907 | 4 | *bi, Doc1, Doc3, Doc2* |
| DNA pol alpha/epsilon subunit B | PF04042 | 2 | *DNApol-alpha73, Pole2* |
| B9 protein | PF07162 | 2 | *tectonic, CG14870* |
