# Supplementary material for: The Gene Regulatory Cascade Linking Proneural Specification with Differentiation in Drosophila Sensory Neurons
Source: PLoS Biol. 2011 Jan 4;9(1):e1000568. doi: 10.1371/journal.pbio.1000568 (PMC3023811; doi:10.1371/journal.pbio.1000568)
Supplement: Table S9 — ato -correlated genes at t3 that have been associated with cilia and/or basal body formation or function and/or are associated with an X box motif. Genes are sorted by overall rank fold-enrichment in atoGFP cells versus the rest of the embryo (>1.5-fold enriched; 1% FDR). (0.20 MB DOC) [file pbio.1000568.s014.doc]

**Table S9**: *ato*-correlated genes at t3 that have been associated with cilia and/or basal body formation or function, and/or are associated with an X box motif.

| **Rank** | **Gene** | **Group or homologue** | **Cilia biogenesis group^1^** | **DCBB^2^** | **X box^3^** | **Other expression evidence^4^** |
| --- | --- | --- | --- | --- | --- | --- |
| 2 | *CG14905* |  | E | + |  |  |
| 9 | *CG18675* |  | D | + |  | B |
| 12 | *CG4525* |  | A | + |  | IS, L, B* |
| 13 | *CG3769* | Dynein LC |  | + | S | IS, L |
| 14 | *tectonic* | B9 domain | A | + | S | AR, L |
| 16 | *CG13125* | LRR |  | + | S | IS |
| 18 | *CG3085* |  |  | + | L | IS, B |
| 19 | *dila* |  | E | + |  | IS |
| 20 | *CG31291* |  |  | + |  | IS, B |
| 21 | *btv* | Dynein |  | + | S |  |
| 22 | *CG10064* |  | D | + |  | B |
| 25 | *CG32703* |  |  | + |  | B* |
| 29 | *CG15161* | IFT46 | A | + | S | IS, AR, L, B |
| 30 | *CG14367* |  | A | + |  | L, B* |
| 32 | *CG18631* |  | A | + |  | L |
| 38 | *CG13617* |  |  | - | L |  |
| 39 | *CG6129* | rootletin |  | + | S | IS, L |
| 40 | *CG30441* | IFT20 | A | + | S | L |
| 43 | *Rfx* |  |  | + |  | IS |
| 49 | *osm-6* | IFT52 | A | + |  | L |
| 51 | *Oseg6* | WDR19 | A | + |  | AR, L |
| 53 | *CG5142* |  | A | + |  | AR, L |
| 55 | *CG14692* |  |  | + |  |  |
| 62 | *CG1126* | BBS5 | A | + | S | AR, L |
| 63 | *CG5964* |  | E | + | L |  |
| 64 | *CG5048* |  |  | + | L | B |
| 67 | *cpo* |  |  | + |  | B, O [1] |
| 68 | *CG6652* |  |  | + |  |  |
| 69 | *Oseg5* | IFT80 | A | + |  | AR, L |
| 70 | *CG6971* |  | C | + |  |  |
| 72 | *osm-1* | Oseg2 | A | + |  | AR, L |
| 74 | *CG16789* |  | E | + |  | B |
| 78 | *CG3259* | Traf3ipI | A | + | S | AR, L |
| 80 | *CG32392* |  | B | + |  | B |
| 84 | *CG6560* | Arl3 | A | + |  | IS, L, B |
| 85 | *sr* |  | A | + |  |  |
| 96 | *nAcRbeta-96A* |  |  | - | L |  |
| 99 | *CG16984* |  |  | + | L | B |
| 101 | *cpo* |  |  | + |  | O [1] |
| 105 | *BBS8* |  | A | - |  | AR, L |
| 108 | *unc* | Basal body |  | - |  | IS, O [2] |
| 109 | *CG17599* |  | A | + |  | L |
| 110 | *Oseg4* | WDR35 | A | + |  | IS, AR, L |
| 112 | *CG14870* |  | A | + |  | AR, L |
| 113 | *Oseg1* | IFT122 | A | + |  | IS, AR, L |
| 117 | *CG6405* |  |  | - | S | B* |
| 132 | *Oseg3* | IFT140 | A | + |  | AR, L |
| 133 | *CG7735/Arl6* | BBS3 | A | + |  | AR, L |
| 134 | *BBS4* |  | E | - |  | AR, L |
| 135 | *Cby* | Wnt signalling |  | - | S |  |
| 140 | *dac* |  | B | + |  | B |
| 141 | *Sulf1* |  |  | + | L | B |
| 143 | *Ets96B* |  |  | + | - | B* |
| 144 | *CG13930* |  |  | + | - |  |
| 150 | *CG8853* | IFT57/  hippi | A | + | S | L |
| 166 | *nompB* |  | A | + |  | L, O [3] |
| 170 | *CG34380* |  |  | - | L |  |
| 173 | *Dhc62B* | Dynein |  | - | L |  |
| 175 | *CG11242* |  | D | + | - |  |
| 181 | *CG8353* |  |  | - | L | IS, B |
| 189 | *CG31036* |  |  | - | S | B |
| 192 | *Wsck* |  |  | - | S |  |
| 194 | *CG9134* |  | E | + | - | B |
| 212 | *CG31321* |  |  | - | S | B |
| 214 | *CG14353* |  |  | + | - |  |
| 217 | *caup* |  |  | + | - |  |
| 227 | *CG13955* |  |  | - | L |  |
| 237 | *Ir* |  |  | + | - |  |
| 262 | *capu* |  |  | + | - | B |
| 263 | *TepII* |  |  | + | L | B |
| 264 | *CG7047* |  |  | - | L |  |
| 273 | *CG31790* |  |  | + | - |  |
| 274 | *CG9170* |  |  | + | - | B* |
| 276 | *pnt* |  |  | - | L | IS, O [4] |
| 295 | *Sp1* |  | A | + | - |  |
| 296 | *nahoda* |  |  | + | - | B |
| 301 | *stan* |  |  | - | L | IS |
| 306 | *CG7724* |  |  | - | L | B |
| 319 | *CG14617* |  |  | + | - | B |
| 321 | *CG4096* |  |  | - | L |  |
| 324 | *Rab23* |  | E | + | - |  |
| 327 | *CG10958* |  | D | + | - | B |
| 331 | *CG2145* |  |  | + | - |  |
| 337 | *CG17387* |  |  | + | - |  |
| 339 | *Dhc93AB* | Dynein |  | + | S |  |
| 345 | *CG5343* |  |  | + | - | B |
| 346 | *RpI12* |  |  | - | L |  |
| 347 | *CG6800* |  |  | + | - |  |
| 352 | *Alh* |  |  | + | - |  |
| 353 | *CG7886* |  |  | - | L |  |
| 354 | *Dhc16F* | Dynein |  | + | - | B* |
| 367 | *CG5195* |  | B | + | - | L |
| 372 | *Hsp23* |  |  | - | L |  |
| 374 | *l(2)05510* |  |  | - | S | B |
| 376 | *Tektin-C* |  |  | - | L | B |
| 384 | *cv-2* |  | B | + | - |  |
| 397 | *Ank2* |  |  | + | - |  |
| 399 | *Ank2* |  |  | + | - |  |
| 400 | *yrt* |  |  | - | S |  |
| 430 | *Klp68D* | Kinesin | E | + | L | B |
| 441 | *shot* |  |  | + | - | O [5] |

Genes are sorted by overall rank fold-enrichment in *ato*GFP cells versus the rest of the embryo (>1.5-fold enriched; 1% FDR).

^1^Cilia biogenesis groups are derived from comparative genomic analysis of ciliated and non-ciliated organisms [6]. The groups are: A. Genes associated with eukaryotes with compartmentalised cilia biogenesis, with few ESTs and nearby X box; B. Genes associated with eukaryotes with compartmentalised cilia biogenesis, with many ESTs or no nearby X box; C. Genes associated with all ciliated eukaryotes; D. Genes associated with eukaryotes that have motile cilia; E. Genes associated with eukaryotes that have prototypical cilia.

^2^DCBB = *Drosophila* Cilia and basal body database [7]. This is compiled from various bioinformatic and proteome sources, although in many cases the evidence is circumstantial. In many cases our data provide the first *in vivo* confirmation of the expression of DCBB genes during cilium formation.

^3^Genes associated with nearby conserved X box; data is from [7]; abbreviations are: S: stringent match (total of 83 genes in the genome); L: loose match (total of 412 genes in the genome). Not all X box-associated genes are currently included in DCBB. These may be new ciliary biogenesis candidates, or they may be involved in other aspects of neuronal differentiation since *Rfx* is known to regulate neuronal differentiation genes other than those involved in ciliary biogenesis [8].

^4^Abbreviations for expression evidence are: IS = *in situ* hybridisation carried out for this study; AR = GFP promoter fusions tested by Avidor-Reiss et al., (2004); L = downregulated by RT-PCR in *Rfx* mutant whole pupal extracts; B = Berkeley *Drosophila* Genome Project database [9] (accessed 1 July 2009) (*=reported in database as ‘no expression’); O = other literature source.

REFERENCES

1. Bellen HJ, Kooyer S, D'Evelyn D, Pearlman J (1992) The *Drosophila* couch potato protein is expressed in nuclei of peripheral neuronal precursors and shows homology to RNA-binding proteins. Genes and Development 6: 2125-2136.

2. Baker JD, Adhikarakunnathu S, Kernan MJ (2004) Mechanosensory-defective, male-sterile unc mutants identify a novel basal body protein required for ciliogenesis in Drosophila. Development 131: 3411-3422.

3. Han Y-G, Kwok BH, Kernan MJ (2003) Intraflagellar Transport Is Required in Drosophila to Differentiate Sensory Cilia but Not Sperm. Current Biology 13: 1679-1686.

4. zur Lage PI, Powell LM, Prentice DR, McLaughlin P, Jarman AP (2004) EGF receptor signaling triggers recruitment of Drosophila sense organ precursors by stimulating proneural gene autoregulation. Dev Cell 7: 687-696.

5. Lee M, Lee S, Zadeh AD, Kolodziej PA (2003) Distinct sites in E-cadherin regulate different steps in Drosophila tracheal tube fusion. Development 130: 5989-5999.

6. Avidor-Reiss T, Maer AM, Koundakjian E, Polyanovsky A, Keil T, et al. (2004) Decoding Cilia Function: Defining Specialized Genes Required for Compartmentalized Cilia Biogenesis. Cell 117: 527-539.

7. Laurençon A, Dubruille R, Efimenko E, Grenier G, Bissett R, et al. (2007) Identification of novel regulatory factor X (RFX) target genes by comparative genomics in Drosophila species. Genome Biology 8: R:195.

8. Kim J, Chung YD, Park D-Y, Choi S-K, Shin DW, et al. (2003) A TRPV family ion channel required for hearing in *Drosophila*. Nature 424: 81-84.

9. Tomancak P, Berman B, Beaton A, Weiszmann R, Kwan E, et al. (2007) Global analysis of patterns of gene expression during Drosophila embryogenesis. Genome Biol 8: 1465.
