# Supplementary material for: The Gene Regulatory Cascade Linking Proneural Specification with Differentiation in Drosophila Sensory Neurons
Source: PLoS Biol. 2011 Jan 4;9(1):e1000568. doi: 10.1371/journal.pbio.1000568 (PMC3023811; doi:10.1371/journal.pbio.1000568)
Supplement: Table S10 — Expression patterns of ato -correlated genes. A summary of patterns observed from in situ hybridization carried out for this study. (0.10 MB DOC) [file pbio.1000568.s015.doc]

**Table S10.** Expression patterns of *ato*-correlated genes.

| **Gene** | **FC^1^ (t1)** | **FC (t2)** | **FC (t3)** | ***in situ* pattern^2^** | **Expression class** | **Notes^3^** |
| --- | --- | --- | --- | --- | --- | --- |
| *CG32150* | 17.39 | 11.80 | 5.94 | All SOPs early, then all type I neurons | Pan-sensory |  |
| *dila* | 11.07 | 17.73 | 9.25 | Ch strong, ES very weak | Ch enriched |  |
| *Rfx* | 9.90 | 8.18 | 2.99 | ato-specific early, pan-sensory later | Ch enriched |  |
| *unc* | 9.76 | 8.10 | 4.97 | All SOPs and progeny | Pan-sensory |  |
| *Esn* | 8.02 | 6.60 | 4.12 | PNS | Pan-sensory |  |
| *CG5597* | 7.22 | 8.59 | 0.90 | All sensory cells, but slightly stronger in Ch | Pan-sensory/ | B* |
| *CG15704* | 6.84 | 4.23 | 3.92 | Small subset of PNS cells | PNS subset |  |
| *CG6129 (rootletin)* | 6.71 | 6.01 | 4.26 | ato-specific early, highly ch neuron specific late | Ch enriched | X |
| *CG6486* | 6.50 | 11.19 | 4.63 | P cell then all PNS at mid stages | Midstage pan-sensory |  |
| *CG15161* | 5.55 | 5.44 | 19.72 | Lateral Ch, late st 11 onwards. Weak elsewhere in PNS | Ch enriched | D, B |
| *CR33327* | 4.84 | 5.08 | 2.00 | Single Ch SOP per segment | Ch subset |  |
| *CG5359* | 4.69 | 2.79 | 2.20 | PNS from stage 10 onwards | Pan-sensory | B |
| *Esg* | 4.35 | 6.18 | 2.55 | All PNS | Pan-sensory |  |
| *Ktub* | 4.21 | 1.85 | 1.13 | PNS | Pan-sensory |  |
| *CG10160 (Imp3)* | 4.13 | 5.58 | 5.47 |  |  |  |
| *CG6560 (Arl3)* | 3.86 | 2.30 | 1.77 | All type I neurons at st 15, subset of PNS earlier but not ch at st 12/13 | Pan-sensory | D, B, X |
| *CG8353* | 3.66 | 3.57 | 1.84 | All type I neurons st 11 onwards | Pan-sensory | D, B, X |
| *CG2069 (Oseg4)* | 3.62 | 3.69 | 2.03 | All type I from 11 onwards but stronger in Ch | Ch enriched | D, B, X |
| *CG16700* | 3.56 | 3.58 | 2.25 | No pattern |  |  |
| *CG11354 (Lim1)* | 2.95 | 10.38 | 1.74 | ato-like at st 11, widespread in PNS later | Ch enriched |  |
| *CG11895 (Stan)* | 2.79 | 2.80 | 2.95 | PNS at st 12 | Pan-sensory |  |
| *CG33182* | 2.74 | 2.88 | 1.29 | Unclear |  | B |
| *CG4525* | 2.53 | 2.36 | 1.08 | All type I neurons at late stage but strongest in lch5 at st 11 | Ch enriched | D, B*, X |
| *CG7161 (Oseg1)* | 2.37 | 3.85 | 3.58 | All type I neurons at late stage but strongest in lch5 at st 11 | Ch enriched | D, X |
| *CG32447* | 1.86 | 2.63 | 6.93 | Tracheal pit ectoderm? |  |  |
| *CG8779 (nrm)* | 1.84 | 1.93 | 2.89 | All SOPs from st 10/11 onwards | Pan-sensory |  |
| *CG32458* | 1.70 | 2.71 | 9.35 | All SOPs from st 10/11 onwards | Pan-sensory |  |
| *fd3f* | 1.60 | 3.84 | 25.74 | ato specific early, ch neuron specific late | Ch specific |  |
| *al* | 1.54 | 4.12 | 9.78 | An early subset of Ch SOPs | Ch subset | B |
| *spdo* | 1.49 | 1.83 | 1.51 | All PNS and CNS, including SOPs | Pan-neural | B |
| *CG3085* | 1.43 | 2.43 | 10.50 | Ch st 11 to 16 | Ch specific | B |
| *CG13889* | 1.42 | 1.33 | 1.48 | PNS, eye st 12 to 14 | Pan-neural |  |
| *CG10339* | 1.37 | 1.97 | 16.41 | Ch, st 13 to 16 | Ch specific |  |
| *CG17564* | 1.32 | 2.23 | 18.41 | Ch st 13 to 16 | Ch specific |  |
| *CG31291* | 1.26 | 1.86 | 4.00 | Ch, weak other PNS, eye st 14 to 16 | Ch enriched | B |
| *CG11253* | 1.24 | 2.21 | 8.77 | Ch, other PNS st 14 to 15 | Ch enriched |  |
| *CG14253* | 1.24 | 1.36 | 7.55 | PNS, eye st 14 | Pan-sensory | B |
| *CG6980* | 1.20 | 2.17 | 8.25 | Ch st 14 to 15 | Ch enriched | B |
| *CG3769* | 1.06 | 0.93 | 0.73 | Ch, weak other PNS st 11 to 16 | Ch enriched |  |
| *CG13125* | 0.84 | 0.99 | 1.70 | Ch st 13 to 15 | Ch specific |  |
| *CG5343* | 0.81 | 1.00 | 9.26 | Ch st 13 onwards | Ch specific | B |
| *CG11382* |  |  |  | PNS st 12 onwards, stronger in Ch | Ch enriched |  |
| *CG31670* | 21.26 | 11.45 | 1.43 | Bolwig’s Organ (larval eye) | Other *ato*-related |  |

^1^FC = fold change (enrichment in *ato*GFP cells versus the rest of the embryo).

^2^*In situ* pattern: a summary of pattern observed from *in situ* hybridization carried out for this study. ^3^Notes: other relevant data: B = BDGP expression images exist (*=no staining previously detected); D = present in DCBB database; X = possesses conserved X box motif in vicinity
