# Supplementary material for: The Gene Regulatory Cascade Linking Proneural Specification with Differentiation in Drosophila Sensory Neurons
Source: PLoS Biol. 2011 Jan 4;9(1):e1000568. doi: 10.1371/journal.pbio.1000568 (PMC3023811; doi:10.1371/journal.pbio.1000568)
Supplement: Table S11 — Genes differentially expressed at t1 in ato GFP cells from wild-type but not in ato mutant embryos. A table of genes that meet the following criteria: ≥2-fold differentially expressed in atoGFP cells from wild-type embryos (fc = ratio of expression in atoGFP cells versus the rest of the embryo) and <2-fold differentially expressed in atoGFP cells from ato-mutant embryos (versus the rest of the embryo) (1% FDR). (0.11 MB DOC) [file pbio.1000568.s016.doc]

**Table S11.** Genes differentially expressed at t1 in *ato*GFP cells from wild-type but not in *ato* mutant embryos.

| **Symbol** | **Gene name** | **Flybase ID** | **FC (t1)** |
| --- | --- | --- | --- |
| *cato* | *cousin of atonal* | [FBgn0024249](http://flybase.org/reports/FBgn0024249) | 16.95 |
| *dila* | *dilatory* | [FBgn0033447](http://flybase.org/reports/FBgn0033447) | 11.07 |
| *unc* | *uncoordinated* | [FBgn0003950](http://flybase.org/reports/FBgn0003950) | 9.90 |
| *Rfx* | *Rfx* | [FBgn0020379](http://flybase.org/reports/FBgn0020379) | 9.76 |
| *ImpL3* | *Ecdysone-inducible gene L3* | [FBgn0001258](http://flybase.org/reports/FBgn0001258) | 7.22 |
| *CG9095* | *CG9095* | [FBgn0030617](http://flybase.org/reports/FBgn0030617) | 5.71 |
| *fd3F* | *forkhead domain 3F* | [FBgn0061173](http://flybase.org/reports/FBgn0061173) | 5.55 |
| *CG30427* | *CG30427* | [FBgn0043792](http://flybase.org/reports/FBgn0043792) | 4.40 |
| *CG15704* | *CG15704* | [FBgn0034103](http://flybase.org/reports/FBgn0034103) | 4.35 |
| *nvy* | *nervy* | [FBgn0005636](http://flybase.org/reports/FBgn0005636) | 4.22 |
| *CG6129* | *CG6129* | [FBgn0039152](http://flybase.org/reports/FBgn0039152) | 4.13 |
| *spdo* |  | [FBgn0011716](http://flybase.org/reports/FBgn0011716) | 3.66 |
| *CG8353* | *CG8353* | [FBgn0032002](http://flybase.org/reports/FBgn0032002) | 3.56 |
| *CG41452* | *CG41452* | [FBgn0084015](http://flybase.org/reports/FBgn0084015) | 3.52 |
| *Atet* | *ABC transporter expressed in trachea* | [FBgn0020762](http://flybase.org/reports/FBgn0020762) | 3.45 |
| *CG32037* | *CG32037* | [FBgn0052037](http://flybase.org/reports/FBgn0052037) | 3.23 |
| *CG12374* | *CG12374* | [FBgn0033774](http://flybase.org/reports/FBgn0033774) | 2.92 |
| *vn* | *vein* | [FBgn0003984](http://flybase.org/reports/FBgn0003984) | 2.75 |
| *CG13653* | *CG13653* | [FBgn0039288](http://flybase.org/reports/FBgn0039288) | 2.72 |
| *Cyp12d1-p* | *Cyp12d1-p* | [FBgn0050489](http://flybase.org/reports/FBgn0050489) | 2.72 |
| *Cyp12d1-d* | *Cyp12d1-d* | [FBgn0053503](http://flybase.org/reports/FBgn0053503) | 2.72 |
| *CG33182* | *CG33182* | [FBgn0053182](http://flybase.org/reports/FBgn0053182) | 2.53 |
| *for* | *foraging* | [FBgn0000721](http://flybase.org/reports/FBgn0000721) | 2.51 |
| *CG6330* | *CG6330* | [FBgn0039464](http://flybase.org/reports/FBgn0039464) | 2.47 |
| *CG31464* | *CG31464* | [FBgn0051464](http://flybase.org/reports/FBgn0051464) | 2.46 |
| *CG6560* | *CG6560* | [FBgn0038916](http://flybase.org/reports/FBgn0038916) | 2.37 |
| *rho* | *rhomboid* | [FBgn0004635](http://flybase.org/reports/FBgn0004635) | 2.36 |
| *phm* | *phantom* | [FBgn0004959](http://flybase.org/reports/FBgn0004959) | 2.33 |
| *CG11671* | *CG11671* | [FBgn0037562](http://flybase.org/reports/FBgn0037562) | 2.32 |
| *CG30085* | *CG30085* | [FBgn0050085](http://flybase.org/reports/FBgn0050085) | 2.28 |
| *peb* | *pebbled* | [FBgn0003053](http://flybase.org/reports/FBgn0003053) | 2.27 |
| *Hil* | *Hillarin* | [FBgn0050147](http://flybase.org/reports/FBgn0050147) | 2.25 |
| *CG8713* | *CG8713* | [FBgn0033257](http://flybase.org/reports/FBgn0033257) | 2.19 |
| *dve* | *defective proventriculus* | [FBgn0020307](http://flybase.org/reports/FBgn0020307) | 2.18 |
| *CG7755* | *CG7755* | [FBgn0034105](http://flybase.org/reports/FBgn0034105) | 2.18 |
| *CG10440* | *CG10440* | [FBgn0034636](http://flybase.org/reports/FBgn0034636) | 2.18 |
| *CG32529* | *CG32529* | [FBgn0052529](http://flybase.org/reports/FBgn0052529) | 2.18 |
| *Cad96Ca* | *Cad96Ca* | [FBgn0022800](http://flybase.org/reports/FBgn0022800) | 2.17 |
| *CG9801* | *CG9801* | [FBgn0037623](http://flybase.org/reports/FBgn0037623) | 2.17 |
| *CG11136* | *CG11136* | [FBgn0034540](http://flybase.org/reports/FBgn0034540) | 2.16 |
| *knrl* | *knirps-like* | [FBgn0001323](http://flybase.org/reports/FBgn0001323) | 2.15 |
| *loco* | *locomotion defects* | [FBgn0020278](http://flybase.org/reports/FBgn0020278) | 2.15 |
| *CG6424* | *CG6424* | [FBgn0028494](http://flybase.org/reports/FBgn0028494) | 2.15 |
| *mRpL45* | *mitochondrial ribosomal protein L45* | [FBgn0038996](http://flybase.org/reports/FBgn0038996) | 2.15 |
| *grn* | *grain* | [FBgn0001138](http://flybase.org/reports/FBgn0001138) | 2.14 |
| *CG14909* | *CG14909* | [FBgn0038458](http://flybase.org/reports/FBgn0038458) | 2.12 |
| *CG17836* | *CG17836* | [FBgn0038661](http://flybase.org/reports/FBgn0038661) | 2.12 |
| *phyl* | *phyllopod* | [FBgn0013725](http://flybase.org/reports/FBgn0013725) | 2.10 |
| *mld* | *molting defective* | [FBgn0083077](http://flybase.org/reports/FBgn0083077) | 2.10 |
| *argos* | *argos* | [FBgn0004569](http://flybase.org/reports/FBgn0004569) | 2.04 |
