# Supplementary material for: The Gene Regulatory Cascade Linking Proneural Specification with Differentiation in Drosophila Sensory Neurons
Source: PLoS Biol. 2011 Jan 4;9(1):e1000568. doi: 10.1371/journal.pbio.1000568 (PMC3023811; doi:10.1371/journal.pbio.1000568)
Supplement: Table S12 — Potential ato target genes based on genes differentially represented in wild-type versus ato -mutant cells. This table shows a subset of the genes in Table S11, selected based on the following additional criterion: ≥2-fold ratio between wild-type and mutant fold-change values (Wt/mut). Compared with the genes in Table S11, this list removes many genes that do not show a robust expression difference between wild type and mutant (i.e., for which differential expression is just above 2-fold in wild type embryos and just below 2-fold in mutant embryos). It is likely that many ato target genes are likely to be excluded by these stringent criteria, particularly those that are expressed widely in other parts of the nervous system or elsewhere in the embryo. A second factor that limits the number of potential targets identified in this way is that a proportion of Ch neurons still develop in ato mutant embryos due to redundancy with the closely related gene, cato [27]. (0.05 MB DOC) [file pbio.1000568.s017.doc]

**Table S12.** Potential *ato* target genes based on genes differentially represented in wild-type versus *ato*-mutant cells.

| **Rank** | **Symbol** | **Gene name** | **Flybase id** | **Wt fc** | **Mut fc** | **Wt/mut** |
| --- | --- | --- | --- | --- | --- | --- |
| 1 | *cato* | *cousin of atonal* | FBgn0024249 | 16.95 | 1.77 | 9.56 |
| 2 | *dila* | *dilatory* | FBgn0033447 | 11.07 | 1.79 | 6.20 |
| 3 | *unc* | *uncoordinated* | FBgn0003950 | 9.90 | 1.91 | 5.20 |
| 4 | *Rfx* | *Rfx* | FBgn0020379 | 9.76 | 1.75 | 5.59 |
| 5 | *ImpL3* | *Ecdysone-inducible gene L3* | FBgn0001258 | 7.22 | 1.40 | 5.15 |
| 6 | *CG9095* | *CG9095* | FBgn0030617 | 5.71 | 1.77 | 3.23 |
| 7 | *fd3F* | *forkhead domain 3F* | FBgn0061173 | 5.55 | 1.76 | 3.15 |
| 8^1^ | *CG30427* | *CG30427* | FBgn0043792 | 4.40 | 1.52 | 2.89 |
| 9^1^ | *CG30427* | *CG30427* | FBgn0043792 | 4.40 | 1.97 | 2.23 |
| 10 | *nvy* | *nervy* | FBgn0005636 | 4.22 | 1.94 | 2.17 |
| 11 | *CG6129* | *rootletin* | FBgn0039152 | 4.13 | 1.72 | 2.40 |
| 12 | *CG41452* | *CG41452* | FBgn0084015 | 3.52 | 1.72 | 2.05 |

^1^Note that *CG30427* is represented by two probe-sets on the chip.
