# Supplementary material for: The Gene Regulatory Cascade Linking Proneural Specification with Differentiation in Drosophila Sensory Neurons
Source: PLoS Biol. 2011 Jan 4;9(1):e1000568. doi: 10.1371/journal.pbio.1000568 (PMC3023811; doi:10.1371/journal.pbio.1000568)
Supplement: Table S13 — Oligonucleotides used for generation of in situ hybridisation probes, GFP reporter constructs, and gel retardation assays. (0.08 MB DOC) [file pbio.1000568.s018.doc]

**Table S13.** Oligonucleotides used for generation of *in situ* hybridisation probes, GFP reporter constructs and for gel retardation assays.

| **Gene** | **Left primer** | **Right primer^1^** |
| --- | --- | --- |
| *CG31670* | ACACGCACAACGACAAGAAG | AACGTGTCCTGGAGGATCTG |
| *CG32150* | GACGAACTGCGAAACGGCAA | CCTCCAACGTCAGACGCTTT |
| *dila/CG1625* | CACCAATGAGGCATCGACATCGG | gctggattgattgattgtgcactta |
| *Rfx/CG6312* | TGAACCACAGCTTCAACAGC | GCCTCATTCCTGCTTCAGTC |
| *Unc/CG1501* | AGGTTGGAGCACACGGATAC | CCTCATGCTCCTCTTCCTTG |
| *EsnCG12833* | AATAGCTAAATCGCCGCGTA | AGACAAGAAGCCGCAAATGT |
| *CG5597* | GGATCGGTTGAGAAGTCTGG | AAAACAGCCACAGGCTGAAG |
| *CG15704* | AAAAGAGTTGGTCGCCTGTG | TTCGCCACTAGAAAAGTTGC |
| *rootletin/CG6129* | GAAGGCTCAAGTGGAGTTCG | TTGTAGGGCCATTTGTAGCC |
| *CG6486* | TTTTTCGATACCCAGCCAAC | GACACTGCGGGGTATAAACG |
| *CG15161* | CAGGAAAACGGTGAAAGGAA | ATTATTCCCACGCCTGACTG |
| *tal/CR33327* | CAGGATCGCAGTCTGAGAA | TCCGTTAGGCGACGTCATC |
| *CG5359* | GTACGGCAGATATGTTCATAG | atttcaggcccatgactgag |
| *esg/CG3758* | CGTTTGGTATTTGTGCATCG | TAGTTTCGGGCGACATATCC |
| *King-tubby/CG9398* | AGATCAATCGACCCATGTCC | CCACGATGTTGTCCATGTTC |
| *ImpL3/CG10160* | GCCGCCATTAAGGACAGTCT | AGCTCGTTCCACTTCTCT |
| *Arl3/CG6560* | TGGCAAGACCACGATACTGA | TTAGTCCAGTGCCATCCAC |
| *CG8353* | AGAACTTCTGACAGCTGCTTTTC | TAGAGGCAGCAAGGGGTTG |
| *Oseg4/CG2069* | GAGGATGCCAAGCAGTTCAT | AGCCAGCACGTACAGCTTC |
| *CG16700* | CTTCGTACCCGTTCTCCTCA | GTCAAACTTCCTCCGACC |
| *lim1/CG11354* | CGAGAATTTTTGCCGTTGTT | TATTGTGCTGGCTGGTGT |
| *stan/CG11895* | ACAACGCCGCCATTAGATAC | ATGACTGGGGCATCTTCAAAC |
| *CG33182* | AGATCGAAGCACCCTAGCTG | ATGGCAAAGTTGGTGGACTC |
| *CG4525* | ATCAGTGGATTGCCTTTTGC | GCGAAGTTGGCTGTAGTTC |
| *Oseg1/CG7161* | GCAACGTCTTCAGTGTCCAA | TGACGCTGTTTACACCCG |
| *CG32447* | AGTAGCCGTAATGCCACCAG | GAGAAGAGCAGCTCCGAGAA |
| *nrm/CG8779* | ATTTCGTTGGCTACGCGGCG | CAACACTGATAGCATGC |
| *CG32458* | CCCTCCGCTACTGACGATAG | CTACATTCGCGCTTTCAA |
| *fd3f/CG12632* | GGCTGAAATCCAAAACCAAC | GGTTGAACTCGTCGCTGAAG |
| *al/CG3935* | CACATGGAATGGCTGGTATG | CATGTCCATTCTGACGCAAC |
| *Dll/CG3629* | CCACAACCACCTCATCCAG | CTCTCGCTCGCTTCTTTCTC |
| *spdo/CG1539/31020* | GAGCAACATGGAATTGAGCA | TTCTGCGGATAGGAGCTGTT |
| *CG3085* | GGCTGGAGAACGTGAAGAAC | CATGCGAGTGAGCGTTATGT |
| *CG13889* | TCGAGAAGCTGGAGGAGAAG | AGCGATTCGTTCAAGGTCTG |
| *CG10339* | CTATGGCCTGATGGACCAGT | ATGTGCTTGTTCGCCTTCTT |
| *CG17564* | GCGACTCCATCGAACTGATA | TCGGATACCTTCACCACCTC |
| *CG31291* | TATCGCCCCAAGAAGAGCTA | TGATCCACCTGGCTGTTGTA |
| *CG11253* | AACAAGCCGGATAAAACGTG | CGGCGCTTAGATGAAACAAT |
| *CG14253* | TGCCCTCCATCGTTATCTTC | TCGCACTTGTTTTCGGTTTT |
| *CG6980* | GCAGTCGAACCAGTCGGTAT | ATCATTGGCATCCTTCTCCA |
| *CG3769* | ATTGGCAGTGGAAAGTCACC | CTCCAATTCGCTCCACACTT |
| *CG13125* | ACAAGCAGGAGCGTGAGATT | agttggccgttagtgtgtgtcc |
| *CAP/CG18408* | agaactccgacgaactggaa | ttggtgtgtgtgtgtgttgg |
| *CG5343* | GGGCCAGTAACTACCAGTCG | GAGTGCTGGGATGGCAGTCTT |
| *GFP* | ccatggtgagcaagggcg | cttgtacagctcgt |
| *atoGFP* construct | ATGTCTAGACAATATTTGCAGAGCCAAG | ATAGGATCCACCACGACGGTCAG |
| *cato1* enhancer | GTGGAGAAGTATTTGTCAG | CTGCACCGACCCGACTTTG |
| *cato2* enhancer | TCCAGGACCAAAGGC | TCATTGCAGATCCGAGCG |
| *fd3F* enhancer | GGCCACAATGAAAGTG | GCACTGCGCTAGTCAG |
| *RfxABC* enhancer | ctaactagcgattgtgatg |  |
| *RfxA* enhancer | ctaactagcgattgtgatg | gcagcgtgacgttttctttc |
| *RfxB* enhancer | aggccagaaagaaaacgtca | tcatttgaatgccgtttg |
| *CG1625* enhancer | tcccataagtcggcacag | ctgccatagcctactggt |
| Gel retardation probes | Top strand: |  |
| *Rfx-*E_ATO_1 | cgagtcctgtgcccaacacatgtttcattatcc |  |
| *dila-* E_ATO_1 | catgcagaggacgcgacacctgttccggcgatcgag |  |
| *dila-* E_ATO_2 | gcagtgctgaccaacatctggcctgctcctgaccctc |  |

^1^In most cases, a T7 RNA polymerase sequence (GTAATACGACTCACTATAGGGC) was added to the right primer for direct digoxygenin-labelled RNA synthesis from the PCR product.
