# Supplementary material for: The Gene Regulatory Cascade Linking Proneural Specification with Differentiation in Drosophila Sensory Neurons
Source: PLoS Biol. 2011 Jan 4;9(1):e1000568. doi: 10.1371/journal.pbio.1000568 (PMC3023811; doi:10.1371/journal.pbio.1000568)
Supplement: Text S1 — FACS isolation of ato GFP cells and validation. (0.04 MB DOC) [file pbio.1000568.s019.doc]

**Text S1. FACS isolation of Ch cells and validation.**

*ato*GFP+ cells were isolated from timed collections of embryos by FACS. Preliminary qRT-PCR and immunohistochemical experiments (not shown) determined that onset of GFP expression, and period of maximal overlap with *ato* expression, occurred at a time point equivalent to 5h45-6h45 of embryo development at 25^o^C. In order to capture the earliest transcriptional events of SOP formation, including those most likely to be direct responses to *ato* activity, embryos aged for this amount formed the first time point for analysis (t1). Additionally, embryos were collected at two further developmental stages in steps of 1 hour (t2: 6h45-7h45; t3: 7h45-8h45). These collections therefore span a range approximately from early stage 11 to late stage 12. Collected embryos were homogenised to give a suspension of single cells. For t1, FACS resulted in identification and isolation of 3% of embryonic cells as being *ato*GFP+ (Figure S1A). This figure is comparable with the numbers of *ato*GFP+ cells anticipated from immunostaining experiments. Analytical FACS demonstrated that the *ato*GFP+ pool was 97% pure. Parallel experiments with non-transgenic (Oregon R) embryos and those expressing GFP ubiquitously confirmed that the cell collection window contained specifically GFP-labelled cells (Figure S1B,C).

Purity and transcriptional integrity were also tested by qRT-PCR. Compared with RNA from whole embryos, *ato* mRNA was 7 times enriched in *ato*GFP+ cells and 10 times depleted in *ato*GFP- cells, given an overall ratio of enrichment of almost 66 (data not shown). The cell sorting takes about 100 min from embryo collection to suspension of cells in RLT buffer. In order to analyse the impact of these incubations, *ato* mRNA levels were measured at different times during the process by qRT-PCR on RNA extracted from sorted cells. This analysis showed that *ato* mRNA levels rose and fell with the same dynamics in whole embryos and in isolated cells of similarly aged embryos (data not shown).
