# Supplementary material for: The Gene Regulatory Cascade Linking Proneural Specification with Differentiation in Drosophila Sensory Neurons
Source: PLoS Biol. 2011 Jan 4;9(1):e1000568. doi: 10.1371/journal.pbio.1000568 (PMC3023811; doi:10.1371/journal.pbio.1000568)
Supplement: Text S2 — Functional gene annotation (GO analysis). (0.04 MB DOC) [file pbio.1000568.s020.doc]

**Text S2. Functional gene annotation (GO analysis)**

We examined the over-representation of gene ontology (GO) annotation terms for genes differentially over-expressed in *ato*GFP+ cells. In order to quantify the over-representation of GO terms we compared the relative GO term frequencies between differentially over-expressed genes and all genes in the genome (Tables S4–6). Functional gene annotations restricted to the GO hierarchy under the GO term GO:0008150 ("biological process") were retrieved directly from the Flybase gene annotation file for all trusted genes and total frequencies calculated by GO term. To determine which GO terms were over-represented in the lists of differentially expressed genes, we first prepared analogous frequency tables for differentially expressed genes. GO terms that were significantly over-represented (p≤0.05) were identified by generating contingency tables from the underlying counts and performing a right-handed corrected Fisher exact test {Hosack, 2003 #1749}. Across all three time points several general PNS related terms were enriched including ‘peripheral nervous system development’ and ‘sensory organ development’, but there were also a large number of terms related to more specialised aspects of PNS development. Interestingly terms are present relating to both visual development (including ‘Bolwig's organ morphogenesis’, ‘compound eye development’, ‘compound eye morphogenesis’, ‘R8 cell fate commitment’) and sensillum development (including ‘sensory perception of sound’, ‘sensory organ precursor cell division’, ‘notum cell fate specification’, ‘ommatidial rotation’, ‘haltere development’ and a number of terms for the Notch signalling pathway). These terms reflect the fact that the *ato*GFP+ pool contains several different *ato*-dependent cells types including both Ch and photoreceptor precursors.

In order to assess systematically the relevance of the over-represented GO terms to PNS development we retrieved all of the GO terms that were associated with genes annotated with the GO term ‘peripheral nervous system development’ (GO:0007422) and compared these terms with the enriched GO term lists. In addition we performed a frequency analysis of the peripheral nervous system related GO terms themselves to determine whether they were over-represented in our enriched GO term lists (Table S7). Overall, for t1, t2, and t3 *ato*GFP+ cells, 56% (79/141), 49.3% (70/142) and 62.3% (71/114) respectively of over-represented GO terms were related to PNS development (Table S7). This represents highly significant enrichments of the order of 2.5–3-fold more than expected by chance.
