# Supplementary material for: The Gene Regulatory Cascade Linking Proneural Specification with Differentiation in Drosophila Sensory Neurons
Source: PLoS Biol. 2011 Jan 4;9(1):e1000568. doi: 10.1371/journal.pbio.1000568 (PMC3023811; doi:10.1371/journal.pbio.1000568)
Supplement: Text S3 — Developmental progression in GO term over-representation. (0.03 MB DOC) [file pbio.1000568.s021.doc]

**Text S3. Developmental progression in GO term over-representation.**

Terms associated with early development (‘Notch signalling pathway’, ‘sensory organ precursor cell fate determination’) decrease from t1 to t3. Conversely, the differentiation terms ‘cilium assembly’ and ‘sensory perception of sound’ increase progressively. Interestingly, ‘DNA replication’ increases at t2 (three of the top eight terms now relate to DNA replication) and then dramatically disappears at t3. This perhaps reflects the change from mitotically quiescent to dividing SOPs, and then towards postmitotic cells (Figure 1E). Overall our experimental approach can detect *in vivo* global gene expression changes over just three hours of neural development. Moreover, expression changes in a manner consistent with progression from neural precursor towards neuronal differentiation.
