# Supplementary material for: The Gene Regulatory Cascade Linking Proneural Specification with Differentiation in Drosophila Sensory Neurons
Source: PLoS Biol. 2011 Jan 4;9(1):e1000568. doi: 10.1371/journal.pbio.1000568 (PMC3023811; doi:10.1371/journal.pbio.1000568)
Supplement: Text S4 — Analysis of ato -correlated genes for over-represented protein domains. (0.04 MB DOC) [file pbio.1000568.s022.doc]

**Text S4. Analysis of *ato*-correlated genes for over-represented protein domains.**

We conducted an analysis of *ato*-correlated genes for over-representation of genes encoding specific protein domains (Table S8, Figure S2). Notably prominent are genes with DNA binding domains (such as homeobox transcription factors), as might be expected since transcriptional regulation is likely to feature highly in the genetic network downstream of *ato*. At later time points, there is a rise in the frequency of domains that may reflect important aspects of the earliest stages of terminal differentiation (e.g. cadherins, ABC transporters, domains associated with protein-protein interactions). Notable over-represented protein families include cadherins and ABC proteins. The cadherins, *E-cadherin* and *shot,* are known to be required for adherens junction development between Ch neurons and their support cells [1]. The novel cadherins may therefore also be involved in the strong cell associations between the cells of the Ch organ. ABC transporters might be involved in secretion into the specialised scolopale lumen surrounding the neuron.

Domain analysis also identified genes associated with ciliogenesis. The B9 domain has been linked to the basal body, which functions to nucleate cilium formation. This group includes *Tectonic*, the vertebrate homologue of which is required for Hh signalling via the primary cilium [2]. The TPR domain is a protein association domain that is particularly linked with Golgi-related vesicle transport and intraflagellar transport (IFT), both processes that are crucial to cilium biogenesis [3]. *ato*-correlated genes of this group include *nompB* (IFT88), *BBS8*, *oseg3* (IFT140), *BBS4* and *oseg6* [4]. BBS4 and BBS8 are part of the BBSome, a protein complex that is required for ciliary membrane extension [5]. NompB, Oseg3 and Oseg6 are part of the IFT complexes. The IFT-B protein complex, along with dynein, is required for transport of materials along the microtubule core into the growing cilium, whereas the IFT-A complex, with kinesin, transports cargo in the opposite direction. Several other TPR genes are enriched that have previously either not been linked with ciliogenesis or not proven to be expressed in sensory neurons.

RFERENCES

1. Lee M, Lee S, Zadeh AD, Kolodziej PA (2003) Distinct sites in E-cadherin regulate different steps in Drosophila tracheal tube fusion. Development 130: 5989-5999.

2. Reiter JF, Skarnes WC (2006) Tectonic, a novel regulator of the Hedgehog pathway required for both activation and inhibition. Genes Dev 20: 22-27.

3. Jékely G, Arendt D (2006) Evolution of intraflagellar transport from coated vesicles and autogenous origin of the eukaryotic cilium. BioEssays 28: 191-198.

4. Avidor-Reiss T, Maer AM, Koundakjian E, Polyanovsky A, Keil T, et al. (2004) Decoding Cilia Function: Defining Specialized Genes Required for Compartmentalized Cilia Biogenesis. Cell 117: 527-539.

5. Nachury MV, Loktev AV, Zhang Q, Westlake CJ, Peränen J, et al. (2007) A Core Complex of BBS Proteins Cooperates with the GTPase Rab8 to Promote Ciliary Membrane Biogenesis Cell 129: 1201-1213.
