# Supplementary material for: The Gene Regulatory Cascade Linking Proneural Specification with Differentiation in Drosophila Sensory Neurons
Source: PLoS Biol. 2011 Jan 4;9(1):e1000568. doi: 10.1371/journal.pbio.1000568 (PMC3023811; doi:10.1371/journal.pbio.1000568)
Supplement: Text S5 — Comparison with proneural cluster-expressed genes from a previous profiling analysis. (0.03 MB DOC) [file pbio.1000568.s023.doc]

**Text S5. Comparison with proneural cluster-expressed genes from a previous profiling analysis.**

A previous expression profiling study has been carried out for the *Drosophila* PNS [1]. In contrast to our study, these authors isolated ectodermal proneural cluster cells from wing imaginal discs (therefore prior to neural commitment). We find a significant but rather modest number of genes to be shared between both studies (Table 1). These shared genes largely comprises genes known to be involved in early neural development. The relatively small overlap in genes found in each analysis is perhaps not surprising since the studies target different neuronal types, time points, and tissues.

1. Reeves N, Posakony JW (2005) Genetic Programs Activated by Proneural Proteins in the Developing Drosophila PNS. Developmental Cell 8: 413-425.
